# Early-Stage Lung Adenocarcinoma MDM2 Genomic Amplification Predicts Clinical Outcome and Response to Targeted Therapy

**DOI:** 10.3390/cancers14030708

**Published:** 2022-01-29

**Authors:** Abhilasha Sinha, Yong Zou, Ayushi S. Patel, Seungyeul Yoo, Feng Jiang, Takashi Sato, Ranran Kong, Hideo Watanabe, Jun Zhu, Pierre P. Massion, Alain C. Borczuk, Charles A. Powell

**Affiliations:** 1 Division of Pulmonary, Critical Care and Sleep Medicine, Icahn School of Medicine at Mount Sinai, New York, NY 10029, USA; abhilasha.sinha@mssm.edu (A.S.); ayushi.patel@nyulangone.org (A.S.P.); feng.jiang@mssm.edu (F.J.); tsato@med.kitasato-u.ac.jp (T.S.); r.kong@stu.xjtu.edu.cn (R.K.); hideo.watanabe@mssm.edu (H.W.); 2 Tisch Cancer Institute, Icahn School of Medicine at Mount Sinai, New York, NY 10029, USA; jun.zhu@mssm.edu; 3 Department of Medicine, Vanderbilt University Medical Center, Nashville, TN 37232, USA; yong.zou@vumc.org (Y.Z.); pierre.massion@vanderbilt.edu (P.P.M.); 4 Perlmutter Cancer Center, NYU Langone Health, New York, NY 10016, USA; 5 Sema4, 333 Ludlow St., Stamford, CT 06902, USA; seungyeul.yoo@sema4.com; 6 Division of Pulmonary Medicine, Department of Medicine, Keio University School of Medicine, Tokyo 160-8582, Japan; 7 Department of Respiratory Medicine, Kitasato University School of Medicine, Sagamihara 252-0374, Japan; 8 Department of Thoracic Surgery, The Second Affiliated Hospital of Medical School, Xi’an Jiaotong University, Xi’an 710004, China; 9 Department of Genetics and Genomic Sciences, Icahn School of Medicine at Mount Sinai, New York, NY 10029, USA; 10 Icahn Institute for Data Science and Genomic Technology, New York, NY 10029, USA; 11 Department of Pathology, Weill Cornell Medicine, New York, NY 10021, USA; alb9003@med.cornell.edu

**Keywords:** early-stage lung adenocarcinoma, MDM2 copy number, tumor heterogeneity, survival, RNA-sequencing, p53, E2F, EMT, therapy response

## Abstract

**Simple Summary:**

Invasive subtypes of lung adenocarcinoma (LUAD) show MDM2 amplification that is associated with poor survival. Mouse double minute 2 (MDM2) is frequently amplified in lung adenocarcinoma (LUAD) and is a negative regulator of p53, which binds to p53 and regulates its activity and stability. Genomic amplification and overexpression of MDM2, together with genetic alterations in p53, leads to genomic and genetic heterogeneity in LUAD that represents a therapeutic target. In vitro assays in a panel of LUAD cell lines showed that tumor cell response to MDM2-targeted therapy is associated with MDM2 amplification.

**Abstract:**

Lung cancer is the most common cause of cancer-related deaths in both men and women, accounting for one-quarter of total cancer-related mortality globally. Lung adenocarcinoma is the major subtype of non-small cell lung cancer (NSCLC) and accounts for around 40% of lung cancer cases. Lung adenocarcinoma is a highly heterogeneous disease and patients often display variable histopathological morphology, genetic alterations, and genomic aberrations. Recent advances in transcriptomic and genetic profiling of lung adenocarcinoma by investigators, including our group, has provided better stratification of this heterogeneous disease, which can facilitate devising better treatment strategies suitable for targeted patient cohorts. In a recent study we have shown gene expression profiling identified novel clustering of early stage LUAD patients and correlated with tumor invasiveness and patient survival. In this study, we focused on copy number alterations in LUAD patients. SNP array data identified amplification at chromosome 12q15 on MDM2 locus and protein overexpression in a subclass of LUAD patients with an invasive subtype of the disease. High copy number amplification and protein expression in this subclass correlated with poor overall survival. We hypothesized that MDM2 copy number and overexpression predict response to MDM2-targeted therapy. In vitro functional data on a panel of LUAD cells showed that MDM2-targeted therapy effectively suppresses cell proliferation, migration, and invasion in cells with MDM2 amplification/overexpression but not in cells without MDM2 amplification, independent of p53 status. To determine the key signaling mechanisms, we used RNA sequencing (RNA seq) to examine the response to therapy in MDM2-amplified/overexpressing p53 mutant and wild-type LUAD cells. RNA seq data shows that in MDM2-amplified/overexpression with p53 wild-type condition, the E2F → PEG10 → MMPs pathway is operative, while in p53 mutant genetic background, MDM2-targeted therapy abrogates tumor progression in LUAD cells by suppressing epithelial to mesenchymal transition (EMT) signaling. Our study provides a potentially clinically relevant strategy of selecting LUAD patients for MDM2-targeted therapy that may provide for increased response rates and, thus, better survival.

## 1. Introduction

Lung adenocarcinoma is associated with a high degree of molecular heterogeneity through multiple mechanisms, such as somatic driver mutations, transcriptional dysregulation, and copy number alterations that include loss or gain in chromosomal region, copy number gain and amplification. In a recent study of 100 TRACERx (Tracking Non–Small-Cell Lung Cancer Evolution through Therapy) [1] tumors, extensive intratumor heterogeneity associated with dynamic copy-number alterations and mutations was reported in NSCLC [2]. The study showed that copy-number heterogeneity was significantly associated with an increased risk of recurrence or death. In LUAD disease, tumor heterogeneity among patients represents an important mechanism driving response and resistance to targeted therapies. The knowledge about how tumor heterogeneity, specifically copy number alterations might alter early LUAD progression and response to therapy remains incompletely defined.

To examine the impact of copy number alterations in early stage LUAD, we examined single-nucleotide polymorphism (SNP) array results on a cohort of LUAD tumors and focused on genome-wide copy number gain. A comprehensive systematic analysis identified copy number (CN) gain in 11–29% of LUAD tumors at chromosome 12q15 on MDM2 locus in different cohorts examined. MDM2 copy number amplification was also found to be associated with high MDM2 protein expression in our LUAD tumors. Notably, tumors with diploid copy number did not display protein overexpression.

MDM2 is a well-known negative regulator of tumor suppressor gene p53 [3]. MDM2 regulates p53 by physically binding to p53, leading to transcriptional deactivation or ubiquitin-mediated degradation [3,4]. Targeting the MDM2–p53 interaction is a potential therapeutic strategy to reactivate p53 function. In the present study, we examined the impact of MDM2 genomic heterogeneity on early lung cancer clinical outcomes and on tumor response to therapy targeting MDM2. This is the first study that suggests MDM2 amplification/overexpression might dictate the patient’s response to MDM2-inhibitor therapy, independent of p53 genetic status. Our finding could be of significance in clinical decision making for treatment of lung adenocarcinoma patients, with the hope of advancing response and survival outcomes.

## 2. Materials and Methods

### 2.1. SNP Array

For SNP array, surgically resected tumor tissue was acquired from Columbia University Medical Center, New York, NY, USA from 1997–2014 (n = 133) all annotated for predominant growth pattern using the WHO classification of lung adenocarcinoma. Additionally, two independent publicly available datasets were downloaded from Broad Institute (n = 553) [5] and TCGA (n = 492) [6]. For SNP array from surgically resected tissue (from Columbia University, New York, NY, USA), DNA from 133 adenocarcinoma patients was extracted from fresh frozen tissue (n = 64) and formalin-fixed paraffin-embedded tissue (n = 69). Fresh frozen tissue was cryostat sectioned at 8μm, stained using 0.1% cresyl violet, and manual needle dissection of tumor-rich areas was performed with an 18 G needle on an Olympus microscope at 40× magnification and collected by vacuum suction into a pipette tip. For each sample, a total of 500 ng of DNA was collected and subjected to DNA copy number analysis using an Affymetrix Genome Wide Human SNP Array 6.0, which includes more than 906,600 SNP probes and >946,000 oligonucleotide probes for the detection of copy number variation (CNV) (Thermo Fisher Scientific, Waltham, MA, USA). Sample preparation, hybridization and scanning were performed using GeneChip^®^ Instrument System according to the manufacturer’s specifications (Thermo Fisher Scientific, Waltham, MA, USA). For FFPE samples, 8 μm sections were de-paraffinized and stained with 0.1% cresyl violet. DNA extraction was performed using Qiagen QIAamp DNA FFPE kit with overnight proteinase K digestions followed by 15 min incubation at 98 °C. A minimum of 80 ng of DNA was provided to Affymetrix Bioinformatics services for sample processing on the Oncoscan FFPE assay.

Data analysis was performed using Nexus Copy Number Software, Version 9.0 (BioDiscovery, Hawthorne, CA, USA) and human NCBI build 36.1). The FASST2 Segmentation algorithm was used to make copy number calls (a Hidden Markov Model approach). The significance threshold for segmentation was set at 1 × 10^−8^, also requiring a minimum of 3 probes per segment and a maximum probe spacing of 1000 between adjacent probes before breaking a segment. The log ratio thresholds for single copy gain and single copy loss were set at +0.1 and −0.15, respectively. The log ratio thresholds for gain of 2 or more copies and for a homozygous loss were set at +0.7 and −1.1, respectively. Array results were analyzed using Nexus Copy Number 9.0 software for subgroup comparisons studies.

### 2.2. Chromogenic In Situ Hybridization (CISH) and Fluorescent In Situ Hybridization (FISH)

CISH was performed on tissue microarrays (n = 443) from Columbia cohort using MDM2 Dual ISH probe cocktails (Ventana Medical systems, Oro Valley, AZ, USA) on an automated Ventana BenchMark XT system (ADRIAMED Ltd., Skopje, North Macedonia) with MDM2 visualized as black signal and CHR12 visualized as red signal. The number of MDM2 and CHR12 signals was counted for 20 nuclei per tumor, and when the ratio was over 2 it is considered amplified. In cases with ratios between 1.8 and 2, 40 cells were counted. Aneuploid cases included those in which ratio was not increased (due to concomitant increase in MDM2 and CHR12 signal) but for which average signal number over 20 cells was over 3. For FISH staining, dual staining was performed using Vysis MDM2/CEP 12 FISH Probe Kit (Abbott, Chicago, IL, USA) with MDM2 (12q15) visualized in orange and centromere of chromosome 12 (12p11.1-q11) visualized in green. An MDM2 and CHR12 signal was counted for 20 nuclei per tumor and quantification of MDM2 signal was conducted similarly to CISH, as detailed above.

### 2.3. Patient Data Analysis for MDM2 and p53 Heterogeneity Distribution

The data from 2 independent LUAD patient cohorts was used to validate the distribution of their MDM2 copy number and p53 mutation. Cohort 1, n = 111 [7] was from resected tumor obtained from Columbia University Medical Center, NY for which p53 status was known. MDM2 CISH was performed on n = 443; however, p53 mutation information was available for n = 111 patients which was for this analysis. Cohort 2, n = 183 [8] was from publicly available LUAD dataset from Broad Institute, MA, USA. For cohort 1 MDM2, copy number was determined by CISH/FISH staining (see methods above). Heat map was drawn by a “heatmap.2” function in the “gplots” package using R.

### 2.4. Immunohistochemistry

Immunohistochemistry for MDM2 was performed on the tissue microarrays (n = 124) from the Columbia cohort using MDM2 antibody (clone 1F2; dilution 1:50; Calbiochem, San Diego, CA, USA) with citrate pH 6 antigen retrieval using a Ventana Benchmark autostainer(Roche, Basel, Switzerland). Tumors were scored as 0, 1, and 2+ staining.

### 2.5. Survival Analysis

Survival analysis was performed on all stages (n = 415) and stage 1 + 2 (n = 354) for cohort from Columbia University Medical Center, NY, USA, for which survival data up to December 2014 was available. MDM2 amplification was as determined by FISH/CISH data. Survival plots were generated using Prism 9 (Graph pad, San Diego, CA, USA).

### 2.6. Cell Culture

All cell lines were acquired from American Type Culture Collection. Lung adenocarcinoma cell lines NCI-H1792, NCI-H23, NCI-H460, NCI-H2009, NCI-H1975 and NCI-H358 were maintained in RPMI 1640 (Gibco, Waltham, MA, USA) supplemented with 10% FBS, 1% penicillin and 1% streptomycin. A549 cells were maintained in DMEM (Gibco) supplemented with 10% FBS, 1% penicillin and 1% streptomycin. All cells were maintained in 37 °C and 5% CO_2_. All cell lines were regularly tested for mycoplasma using the mycoAlert Detection Kit (Lonza, Basel, Switzerland).

### 2.7. MDM2 Inhibitor

RG7112 (>99% HPLC, Seleckchem, Houston, TX, USA) was used for in vitro assays to treat cell lines. RG7112 was dissolved in DMSO, as per manufacturer’s instructions for making main stock, and was further diluted in media for use in in vitro functional assays.

### 2.8. Migration and Invasion

For migration assay, 50 × 10^3^ cells were seeded with DMSO, 0.1 µM RG7112 or 1 µM RG7112 on an 8 μM cell culture insert (Fisher Scientific, Hampton, NH, USA) in triplicate wells in serum free media in a 24-well plate and 800 μL of 10% FBS supplemented media was added to the lower compartment of the well. Cells were incubated for 48 h in 37 °C incubator and then the inserts were washed with phosphate-buffered saline. Cells on the top of transwell were scraped and washed away using cotton tip applicator. Cells on the bottom side of transwell were fixed with 70% ethanol and stained with 0.2% crystal violet. Images were analyzed using ImageJ software for each replicate.

For invasion assay, 8 μM cell culture insert was coated with 300 μg/mL of Corning matrigel basement membrane matrix (Corning, Corning, NY, USA) and incubated for 1 h in 37 °C incubator. 50 × 10^3^ cells were then seeded with DMSO, 0.1 µM RG7112 or 1 µM RG7112 over the layer of matrigel in the 8 μM cell culture insert and was stained in a similar fashion as migration assay.

### 2.9. Wound Healing Assay

Cells were cultured to 100% confluency in a 24 well plate and wound was carefully created using P20 pipette tip. Cells were treated with DMSO, 0.1 µM RG7112 or 1 µM RG7112 and images of wound were captured at 0 h, 24 h and 72 h to evaluate closure of wound for each treatment group. Images were analyzed using ImageJ software (National Institutes of Health, Bethesda, MD, USA).

### 2.10. Western Blot

Cells were harvested to obtain total protein extract for Western blot. Whole cell protein extract was prepared from cells using RIPA lysis buffer (Thermo Scientific, Waltham, MA, USA). Protein concentration was estimated using Pierce BCA protein assay kit (Thermo Scientific) and 40 μg of protein was boiled in Laemmli’s SDS sample buffer (Boston bioproducts, Ashland, MA, USA) to run on SDS-PAGE gel. Protein was electro-transferred to PVDF membrane, blocked with 5% non-fat powdered milk (Boston bioproducts), followed by overnight incubation with primary antibody at 4 °C. The membrane was washed three times with 0.05% Tris-buffered saline Tween-20 (TBST) wash buffer for 10 min each and incubated with HRP-conjugated secondary antibody. Membrane was then washed three times with TBST and developed with Clarity Western ECL substrate (Bio-Rad Laboratories, Hercules, CA, USA). All the whole western blot figures can be found in the supplementary materials.

### 2.11. Proliferation Assay

For IC_50_ 1–2 × 10^3^ cells were seeded in triplicate wells in a 96-well plate for treatment with serial dilutions of RG7112 (MedChem Express, Monmouth Junction, NJ, USA). Effect of RG7112 on the viability of lung adenocarcinoma cells was tested at 48 h after drug treatment using alamarBlue™ Cell Viability Reagent. IC_50_ value for the effect of drugs on cell viability was calculated by plotting log inhibitor vs. normalized response- variable slope on Prism.

### 2.12. RNA Sequencing and Library Preparation

For RNA sequencing, A549 and H1792 cells were treated with DMSO or 1 μM RG7112 for 48 h and total RNA was purified using Rneasy kit (Qiagen, Hilden, Germany). Using 1 μg of RNA from each sample, poly-A RNA was enriched using the NEBNext PolyA mRNA Magnetic Isolation Module (New England Biolabs, NEB, Ipswich, MA), followed by incubation at 94 °C for 15 min. Double-stranded complementary DNA (cDNA) was synthesized using SuperScript III reverse transcriptase (Thermo Fisher Scientific) and NEBNext Ultra II Directional RNA Second Strand Synthesis Module (NEB). For Illumina sequencing, library construction was performed using up to 10 ng of cDNA using the NEBNext Ultra DNA Library Prep Kit (NEB). Paired-end sequencing was performed on HiSeq 2000 (Illumina, San Diego, CA, USA) for 150 nt from each end according to the manufacturer’s instructions.

### 2.13. RNA-Seq Analysis

Sequencing reads were pseudoaligned to the human reference transcriptome GRCh38.95 from Ensembl and transcript abundance was estimated using kallisto (v0.45.0) (Pachter lab, University of California, Berkeley, CA, USA). [9]. Transcript abundance was aggregated to gene level count using biomaRt (Bioconductor R-package, https://www.bioconductor.org/) (accessed on 20 December 2021) annotation. DEseq2 [10] was used to identify differentially expressed genes between control treated cells and MDM2 inhibitor treated cells for A549 and H1792, respectively. Differentially expressed genes (DEGs) were defined using a cutoff of absolute log2 fold change set at 1 and FDR < 0.05.

### 2.14. Gene Set Enrichment Analysis

To determine underlying potential signaling pathways and transcriptional targets enriched with MDM2 inhibitor (RG7112) treatment, the overlap of downregulated genes in both cell lines was compared with gene sets curated in the Molecular Signatures Database (MSigDB). [11] for “Hallmark gene sets”, “C2 curated gene sets” and “transcription factor targets” collection with FDR < 0.05 for multiple comparisons.

### 2.15. Statistical Analysis

Chi-square test was performed using Prism to determine correlation between MDM2 copy number (CISH/FISH) and protein expression (IHC) in LUAD patients n = 124.

One-way ANOVA multiple comparisons was used to determine significance in transwell migration and invasion assay. Two-way ANOVA multiple comparisons were used to determine significance in wound healing assay.

## 3. Results

### 3.1. MDM2 Genomic Heterogeneity in LUAD Patients

**Figure 1:**
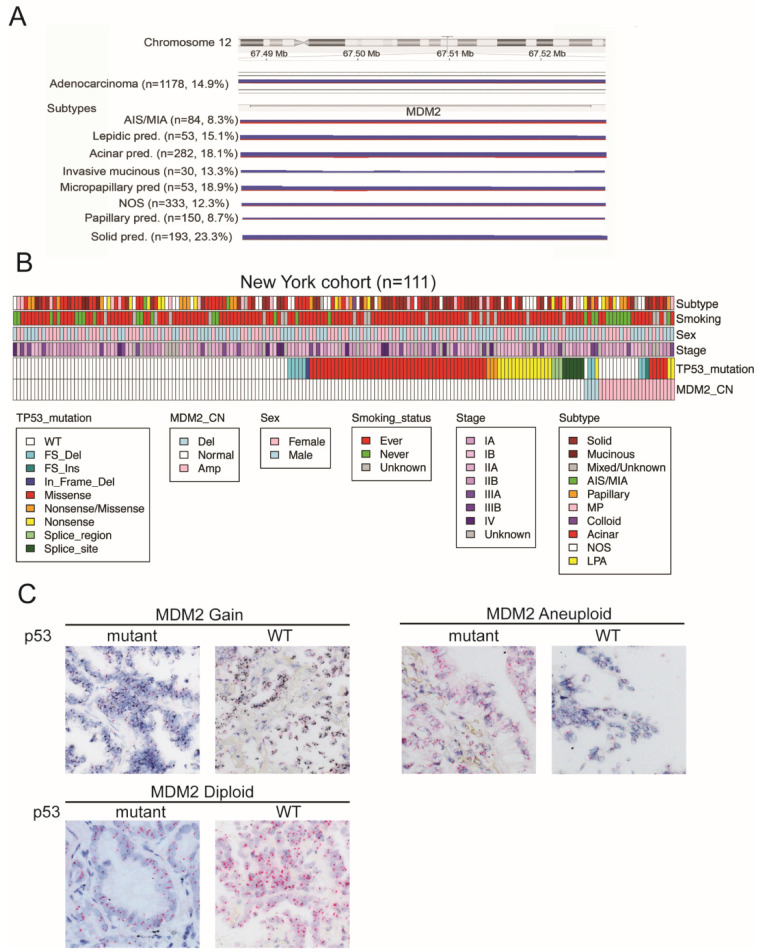
MDM2 genomic amplification and p53 mutation contribute to heterogeneity in lung adenocarcinoma. (**A**) Composite analysis of SNP array data from n = 1178 LUAD patients showing amplification (blue bars) or deletion (red bars) at MDM2 chromosome locus 12q15. Width of blue and red bars indicated the percent of samples in that group displaying MDM2 amplification in SNP array. Percent of samples with MDM2 amplification for total and each subtype is annotated on the left. (**B**) Heatmap showing distribution of MDM2 copy number (CN) and p53 mutation status for n = 111 LUAD patients from New York cohort. MDM2 CN was determined by CISH/FISH analysis. Subtype, smoking status, sex and stage for each patient is shown in top rows. (**C**) Representative images for CISH staining showing MDM2 amplification in MDM2 gain, aneuploid and diploid condition with p53 mutant or WT for each case. MDM2 is visualized as black signal and CHR12 is visualized as red signal. The magnification of the figure is 10×.

We performed a genome-wide SNP array on surgically resected LUAD tumors and performed a composite analysis by merging our own SNP array data (Table S9) with publicly available data from two independent cohorts, one from The Cancer Genome Atlas Program (TCGA) (n = 492) and another from Broad Institute, MA, USA (n = 553). Our SNP array composite analysis included 1178 LUAD cases including 84 Adenocarcinoma in situ (AIS)/Minimally invasive adenocarcinoma (MIA), 53 Lepidic predominant adenocarcinoma (LPA) 282 acinar predominant (AC), 150 papillary predominant (PAP), 193 solid predominant (SOL), 53 micro-papillary predominant (MP), 30 invasive mucinous adenocarcinoma (MUC) and 333 invasive adenocarcinomas in which predominant pattern was not annotated. SNP array composite analysis showed amplification (blue bars) at MDM2 locus in ~15% LUAD cases (Figure 1A and Figure S1A). Subtype breakdown showed MDM2 was amplified in 8% AIS/MIA, 15% LPA, 18% AC, 13% MUC, 19% MP, 8% PAP and ~23% in SOL cases (Figure 1A). This data indicated a higher percentage of MDM2 amplification in LUAD patients with solid, micropapillary and invasive mucinous subtypes that are associated with increased aggressiveness and lower survival [12,13] across all subtypes with the highest in most invasive solid subtype, suggesting MDM2 gain is associated with more tumor invasiveness [14,15].

To validate SNP array-detected MDM2 amplification, we performed FISH/CISH on a LUAD tissue microarray. The FISH/CISH data showed focal amplification of MDM2 in 29% of LUAD patients (Figure 1B,C and Table S1). P53 mutation data was available in a subset of cases (Figure 1B,C and Table S2). Among tumors with MDM2 ampl and diploid status subset, there was a group of patients with p53 WT and another with p53 mutant, respectively. This suggested that MDM2 genomic amplification together with p53 genetic mutation contributes to heterogeneity among LUAD patients.

MDM2 amplification and its association with p53 mutation pattern was examined in two independent cohorts. Cohort 1 was obtained from resected tumors from New York, for which p53 status was known and a second cohort was accessed from the publicly available LUAD dataset from Broad Institute, MA, USA [8]. In the New York cohort (n = 111), among MDM2-amplified cases, 68% were p53 WT and 31% were p53 mutant and among MDM2 diploid cases, 83% were p53 WT and 16% were p53 mutant (Figure 1B and Table S2). In the Broad cohort (n = 183), MDM2 was amplified in 11% of total patients (Table S3). Among MDM2-amplified cases, 52% were p53 WT and 48% were p53 mutant and among MDM2 diploid cases 47% patients had WT p53 and 53% patients had mutant p53 (Figure S1B and Table S3). Together our SNP array and FISH/CISH data and publicly available independent dataset indicated that MDM2 genomic status and p53 mutation contributes to heterogeneity among LUAD patients. It is therefore critical to determine whether this heterogeneity alters patient survival and response to targeted therapy. The knowledge about how MDM2 heterogeneity might influence patient’s therapeutic response could potentially provide benefit for LUAD patients in clinic.

### 3.2. MDM2 Genomic Amplification Is Associated with Protein Overexpression and Poor Survival in LUAD

**Figure 2:**
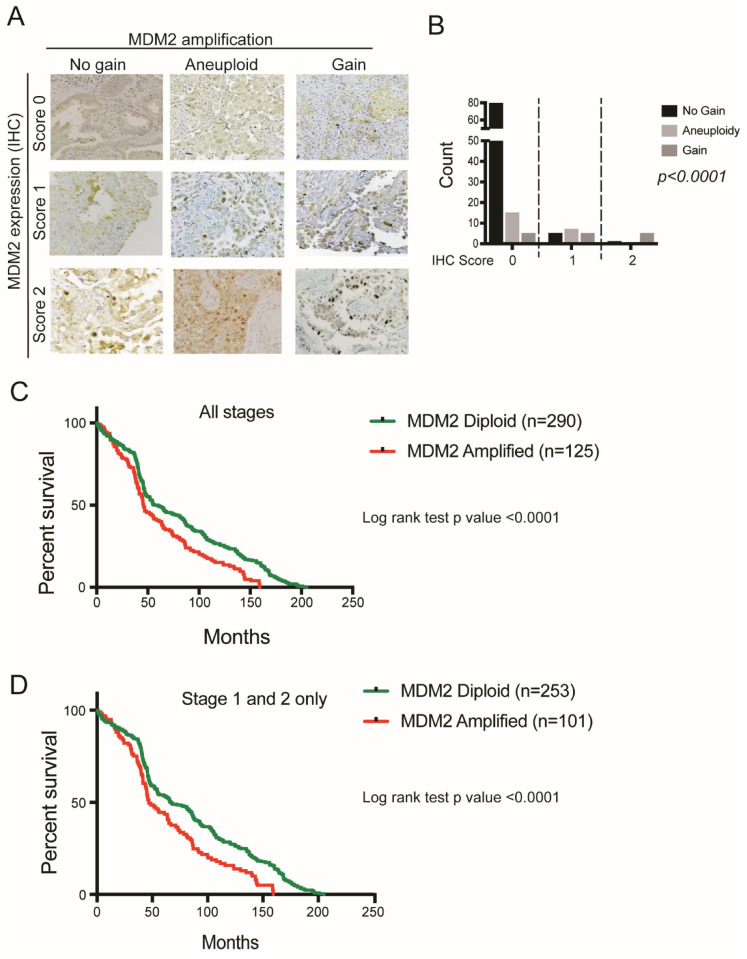
MDM2 copy number amplification is correlated with protein overexpression and worse survival in LUAD patients. (**A**) Representative images for MDM2 immunohistochemistry on LUAD tissue microarray n = 124, for patients with MDM2 IHC score 0, 1 or 2 having no gain, aneuploid and gain as identified by FISH/CISH. (**B**) Chi-square plot showing correlation between MDM2 copy number and IHC protein expression in LUAD patients, n = 124, *p* < 0.0001. (**C**) Percent overall survival for MDM2 amplified (n = 125) and MDM2 diploid (n = 290) LUAD patients for all stages, LRT *p* < 0.0001. (**D**) Percent overall survival for MDM2 amplified (n = 101) and MDM2 diploid (n = 253) LUAD patients for Stage 1 and 2 only, LRT *p* < 0.0001. The magnification of the figure is 10×.

MDM2 amplification was observed in 29% and 11% of cohort 1 and 2, respectively. To investigate the correlation between MDM2 copy number gain and protein expression in LUAD patients, we performed immunohistochemistry for MDM2 expression in LUAD tissue microarray (n = 124). Patients were scored 0, 1 or 2 for their MDM2 protein expression. MDM2 copy number was positively correlated with protein expression (Figure 2A,B). 83% (5/6) patients with IHC score 2 had MDM2 gain while 80% (81/101) patients with IHC score 0 had no gain, showing that MDM2 gain is associated with MDM2 protein overexpression in LUAD patients.

To examine the clinical relevance of MDM2 copy number gain in LUAD patients, we determined if amplification was associated with survival in NY cohort patients. Survival data was available for n = 415 patients. We performed survival analysis on patients for “all stages” (n = 415) as well as “stage 1 + 2” only (n = 354) to avoid confounding by advanced stage disease. Patient survival data showed that patients with MDM2 amplification had inferior survival both in all stages as well as for stage 1 + 2 (Log rank test (LRT) *p* < 0.0001 for both comparisons) (Figure 2C,D).

To best mimic the MDM2 genomic heterogeneity with p53 WT and mutation background as seen in LUAD patients, we chose a panel of seven cell lines out of which four with MDM2 amplification (ampl), with or without p53 mutation, and three cell lines with diploid MDM2 copy number (hereafter, Diploid) with or without p53 mutation (Figure S2A). Copy number data for MDM2 amplification and p53 mutation information for each cell line was downloaded from Cancer Cell Line Encyclopedia (CCLE, Broad Institute). Among the seven cell lines chosen for the study, H1792 and H23 have MDM2 ampl and p53 mutation and A549 and H460 are MDM2 ampl and p53 WT (Figure S2A). For MDM2 diploid condition we used H1975 and H2009 with p53 mutation and H358 cells with p53 WT (Figure S2A). Consistent to our LUAD patient data, our Western blot analysis on panel of seven cell lines showed that all four cell lines with MDM2 ampl (H1792, H23, A549, and H460) displayed significantly higher MDM2 protein expression as compared to cell lines with diploid MDM2 (H1975, H2009 and H358) (Figure S2B). These data suggested that MDM2 gain is correlated with higher protein expression both in LUAD patients and cell lines.

### 3.3. Anti-Migratory and Anti-Invasive Effect of MDM2 Inhibitor in MDM2-Amplified Tumor Cells

Lung adenocarcinoma pathology and biology is heterogeneous, which is reflected by tumor MDM2 amplification status (Figure 1). MDM2 is amplified in p53 wild-type (WT) as well as mutant condition (Figure 1B,C). We investigated the role of MDM2 status on response to MDM2 inhibitor RG7112 on migratory and invasive ability of lung adenocarcinoma cells using in vitro transwell migration and invasion assays.

**Figure 3:**
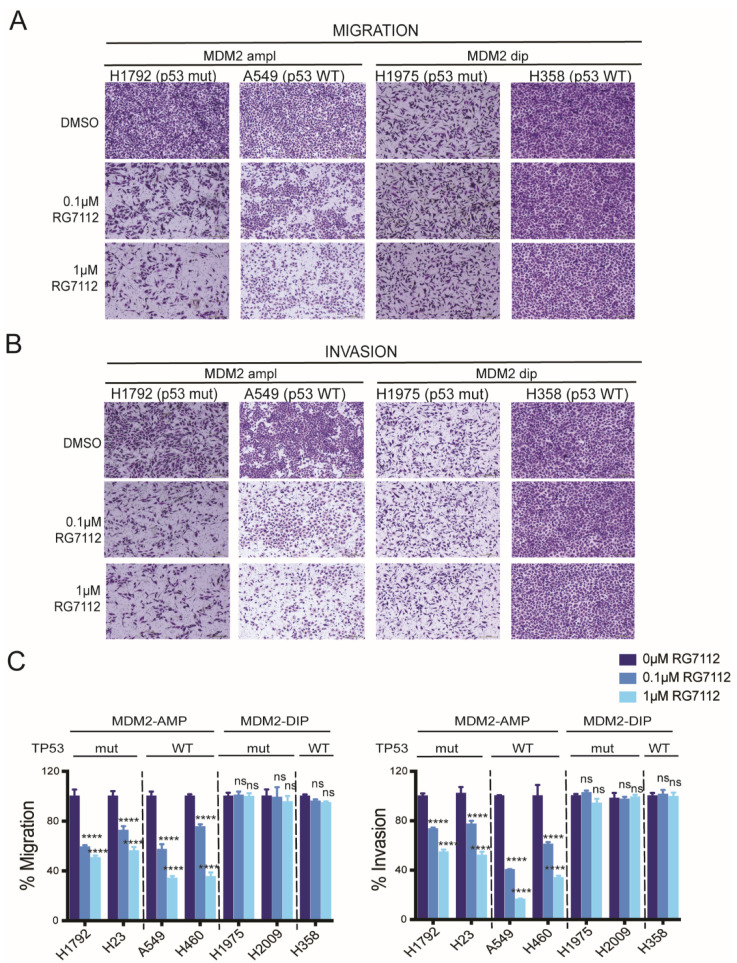
Anti-migratory and anti-invasive effect of MDM2 inhibitor shows MDM2-dependent drug response. (**A**) Representative images of transwell migration assay for H1792 (MDM2 ampl; p53 mutant), A549 (MDM2 ampl; p53 WT), H1975 (MDM2 dip; p53 mutant) and H358 (MDM2 dip; p53 WT group) on treatment with DMSO, 0.1 µM RG7112 or 1 µM RG7112 for 48 h. (**B**) Representative images of transwell invasion assay for H1792 (MDM2 ampl; p53 mutant), A549 (MDM2 ampl; p53 WT), H1975 (MDM2 dip; p53 mutant) and H358 (MDM2 dip; p53 WT group) on treatment with DMSO, 0.1 µM RG7112 or 1 µM RG7112 for 48 h. (**C**) Quantitation of % Migration (left) and % Invasion (right) for panel of 7 cell lines on treatment with DMSO, 0.1 µM RG7112 or 1 µM RG7112 for 48 h. MDM2 ampl and p53 mutation annotations are labelled on the top. One-way ANOVA, *p*-values for each comparison in results. **** *p* < 0.0001 and ns non significant. The magnification of the figure is 4×.

In transwell migration assays, cells with MDM2 amplification displayed significantly reduced migration regardless of p53 mutation status (*p* < 0.0001 for H1792, H23, A549 and H460 for all comparisons, Figure 3A,C). On the other hand, all 3 LUAD cell lines with diploid MDM2 copy number showed no effect on migration with drug treatment (H1975 *p* = 0.9897 and *p* = 0.9988, H2009 *p* = 0.979 and *p* = 0.639, H358 *p* = 0.7167 and *p* = 0.5717 for 0 μM vs. 0.1 μM and 0 μM vs. 1 μM RG7112 treatment for each cell line, respectively) (Figure 3A,C).

We next evaluated whether LUAD cells display differences in invasiveness response to MDM2 inhibitor. The in vitro transwell matrigel invasion assay similarly showed a significant reduction in invasiveness of LUAD cells in an MDM2 dependent manner. LUAD cells with MDM2 amplification showed suppressed invasiveness in response to MDM2 inhibitor, irrespective of p53 status (*p <* 0.0001 for H1792, H23, A549 and H460 for all comparisons) (Figure 3B,C). LUAD cells with diploid MDM2 did not respond to the drug treatment (H1975 *p* = 0.8508 and *p* = 0.4008, H2009 *p* = 0.9851 and *p* = 0.9694, H358 *p* = 0.9527 and *p* = 0.9912 for 0 μM vs. 0.1 μM and 0 μM vs. 1 μM RG7112 treatment for each cell line, respectively) (Figure 3B,C). The migration and invasion assay data indicate that response to MDM2-inhibitor therapy is dependent on MDM2 genomic amplification status; this suggests that MDM2 inhibitor efficacy will be highest in tumors with MDM2 amplification, compared to those with no MDM2 gain, irrespective of their p53 genetic status. This suggests that the inter-patient MDM2 heterogeneity within LUAD patients is a critical aspect to consider for targeting patients who may benefit from the MDM2-inhibitor therapy.

### 3.4. MDM2 Inhibitor Suppresses Cell Motility in MDM2 Dependent Manner

**Figure 4:**
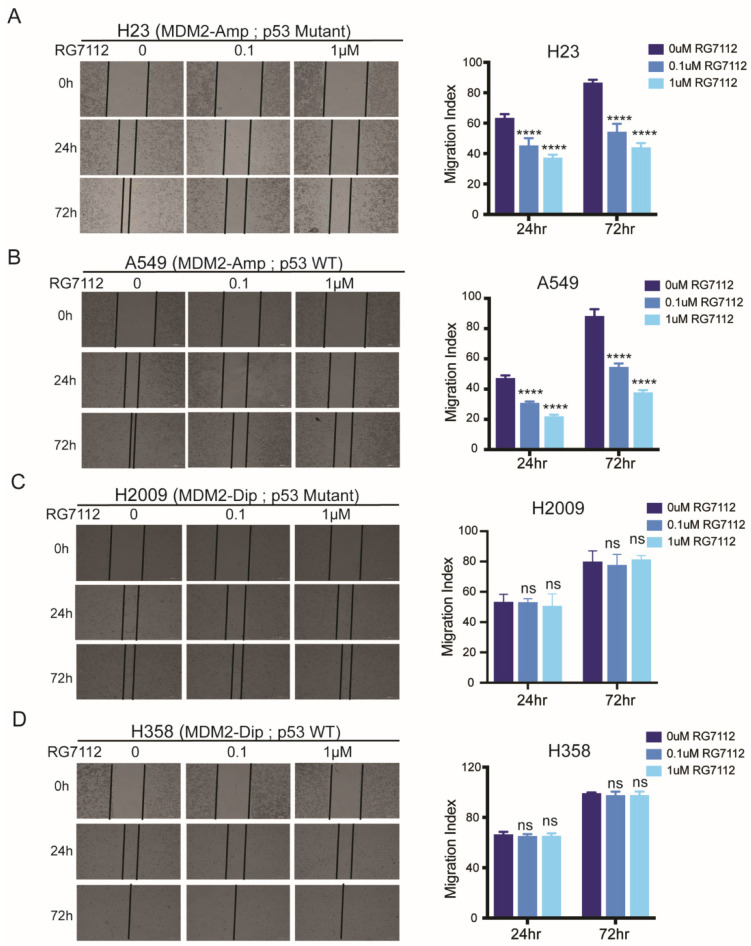
MDM2 inhibitor suppresses cell motility in MDM2 dependent manner. (**A**–**D**) Representative images (left) and quantitation of Migration index (right) for wound healing assay for (**A**) H23 (MDM2 ampl, p53 mutant), (**B**) A549 (MDM2 ampl, p53 mutant), (**C**) H2009 (MDM2 dip, p53 mutant) and (**D**) H358 (MDM2 dip, p53 WT), treated with DMSO, 0.1 µM RG7112 or 1 µM RG7112 imaged at 0 h, 24 h and 72 h, respectively. Two-way ANOVA, *p*-values for each comparison in Table S4. **** *p* < 0.0001 and ns non significant.

We evaluated response to MDM2-inhibitor therapy using in vitro wound healing assays. LUAD cells with MDM2 amplification showed significant suppression in cell motility with RG7112 treatment, irrespective of p53 mutation status (H23: *p* < 0.0001 for all comparisons at 24 h and 72 h, A549: *p* = 0.0002 and *p* < 0.0001 for 0 μM vs. 0.1 μM and 0 μM vs. 1 μM RG7112 treatment at 24 h, respectively, and *p* < 0.0001 for all comparisons at 72 h) (Figure 4A,B). Conversely, diploid MDM2 cell lines did not show effect on cell motility in response to the drug treatment, *p* values in Table S4 (Figure 4C,D).

The impact on migration, invasion and wound healing was independent of cell proliferation which was mildly decreased at high concentration of RG7112 (IC50 ~9–10 μM) (Figure S3A,B). The inhibitory effect in suppressing migration, invasion and wound healing ability was significantly higher at a much lower drug concentration (0.1 μM and 1 μM), suggesting the effect of the drug to be more pronounced in suppressing migration/invasion ability of lung adenocarcinoma cells.

### 3.5. MDM2 Inhibitor Alters Unique Signaling in MDM2 Amplified; p53 Mutant vs. WT Background

**Figure 5:**
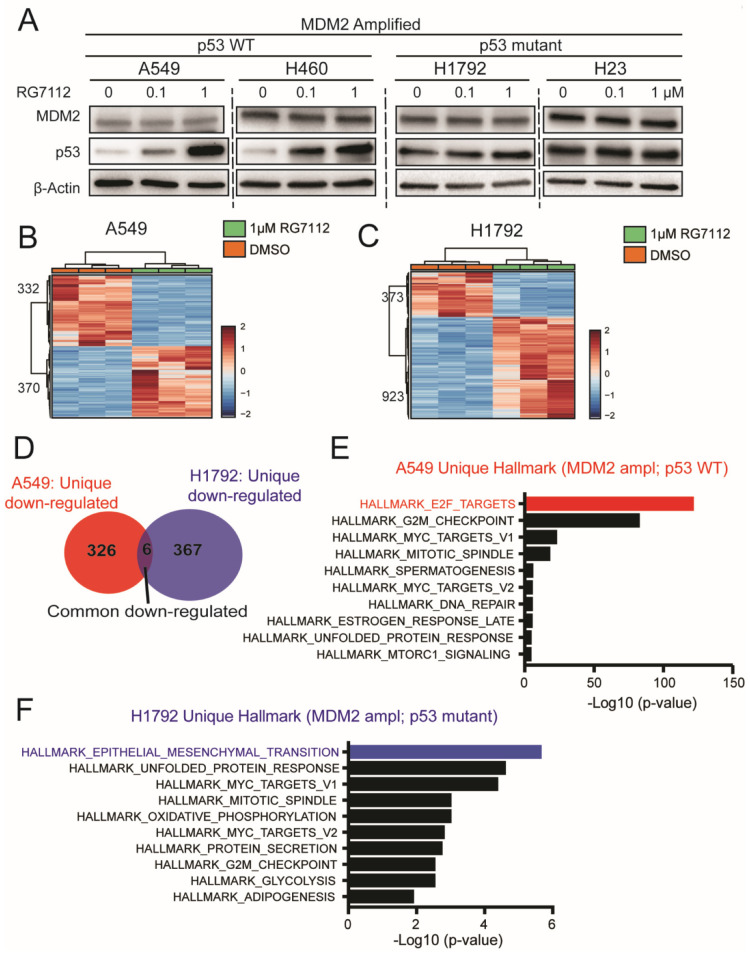
MDM2 inhibitor alters unique signaling in MDM2 amplified; p53 mutant vs. WT background. (**A**) Western blot for MDM2, p53 and β-actin in A549, H460 (MDM2 ampl; p53 WT) and H1792, H23 (MDM2 ampl; p53 mutant) treated with DMSO, 0.1µM RG7112 or 1µM RG7112 for 48 h. (**B**,**C**) Heatmap showing DEGs with log2 fold change greater or less than 1 and FDR < 0.05 (**B**) A549 (MDM2 ampl; p53 WT) and (**C**) H1792 (MDM2 ampl; p53 mutant) treated with DMSO or 1 µM RG7112 for 48 h. (**D**) Venn diagram showing unique and overlapping differentially expressed downregulated genes in A549 (MDM2 ampl; p53 WT) and H1792 (MDM2 ampl; p53 mutant) on treatment with 1 µM RG7112. (**E**,**F**) Hallmark enrichment analysis for differentially expressed downregulated unique genes in (**E**) A549 (MDM2 ampl; p53 WT) and (**F**) H1792 (MDM2 ampl; p53 mutant) on treatment with 1 µM RG7112.

Our in vitro functional and phenotypic data revealed suppression in migration and invasion in response to MDM2 inhibitors in MDM2-dependent manner, in both p53 WT and mutant background. RG7112 is a member of the nutlin family of MDM2 inhibitors which binds in the p53-binding pocket of MDM2 and blocks its interactions with p53 [16]. As expected, our Western blot data displayed restoration of p53 protein in a dose dependent manner with RG7112 treatment only in p53 WT cells A549 and H460 cell lines (Figure 5A). Importantly, the MDM2 inhibitor suppresses tumorigenic features of LUAD cells in both p53 WT as well as mutant background (Figure 3 and Figure 4). To determine biological mechanisms underlying this response, we performed RNA seq on A549 (MDM2 ampl; p53 mutant) and H1792 (MDM2 ampl; p53 WT) cells treated with 1 μM RG7112. As expected, RNA seq showed a distinct pattern of differentially expressed genes (DEG) in supervised clustering and principal component analysis (Figure 5B,C) (Figure S4A–D) for both cell lines. DEGs between the two groups were determined based on t-test FDR < 0.05 and log_2_ fold-change >1 for up-regulated genes and <−1 for downregulated genes, with a total of 702 and 1296 DEGs in A549 and H1792, respectively. To understand the downstream signaling responsible for therapy response, we focused our analysis on DEGs downregulated by RG7112 treatment. A total of 332 and 373 DEGs were downregulated in A549 and H1792 with RG7112 treatment, respectively. Gene set analysis identified only six common genes between A549 and H1792 downregulated DEGs, while most genes were unique (Figure 5D, Table S5), suggesting that MDM2 inhibitor impacts distinct mechanisms in the two different genetic background model systems to suppress tumorigenic features.

To identify signaling pathways through which MDM2 inhibitors suppress migration and invasion in p53 mutant and WT genetic background, we functionally annotated the unique genes downregulated with RG7112 treatment. We performed gene ontology (GO) analysis on 326 and 367 unique downregulated DEGs (Figure 5D) using Hallmark gene sets, C2 curated gene sets and transcription targets curated in the MSigDB. Hallmark gene set enrichment analysis identified E2F targets as most enriched in MDM2 ampl; p53 WT A549 cells (Figure 5E, Table S6). Interestingly, Hallmark gene set analysis revealed enrichment of Epithelial Mesenchymal Transition (EMT) signaling in MDM2 ampl; p53 mutant H1792 cells as the top enriched downregulated pathway (Figure 5F, Table S7).

C2 curated gene set analysis in MDM2 ampl; p53 WT A549 cells showed enrichment of E2F targets and DREAM complex [17], which is an E2F regulated cell cycle regulating complex (Figure S5A, Table S6). These data suggest that in MDM2-amplified and p53 wild-type LUAD conditions, MDM2-targeted therapy suppresses tumor invasion through altering E2F signaling. The C2-curated gene set analysis revealed enrichment of pathways involved in “haptotaxis” [18,19] and “chemotaxis” [20,21] in MDM2 ampl; p53 mutant H1792 cells (Figure S5B, Table S7), validating the impact on migratory-related signaling in p53 mutant condition.

The GO analysis for the transcription-target gene sets downregulated with RG7112 treatment was significantly enriched for E2F target gene sets in MDM2 ampl; p53 WT A549 cells (Figure S5C, Table S6). On the contrary, in MDM2 ampl; p53 mutant H1792 cells we observed a distinct set of transcriptional targets including FXR1 [22,23,24], NUP153 [25,26], SFMBT1 [27,28] and THAP1 [29], which are known to be involved in cell motility and invasion processes (Figure S5D, Table S7), suggesting distinct pathways involved in response to therapy in the two different p53 genetic background in MDM2 ampl LUAD. Together these data suggested that MDM2 inhibitor suppresses tumor migration and invasion in p53 mutant and WT genetic background by distinct mechanisms. The MDM2 inhibitor suppresses migration and invasion in p53 WT condition through targeting E2F while in p53 mutant condition it targets EMT signaling.

### 3.6. MDM2-Targeted Therapy Suppresses Tumorigenesis in p53 Wild-Type Condition through E2F → PEG10 → MMP Signaling

**Figure 6:**
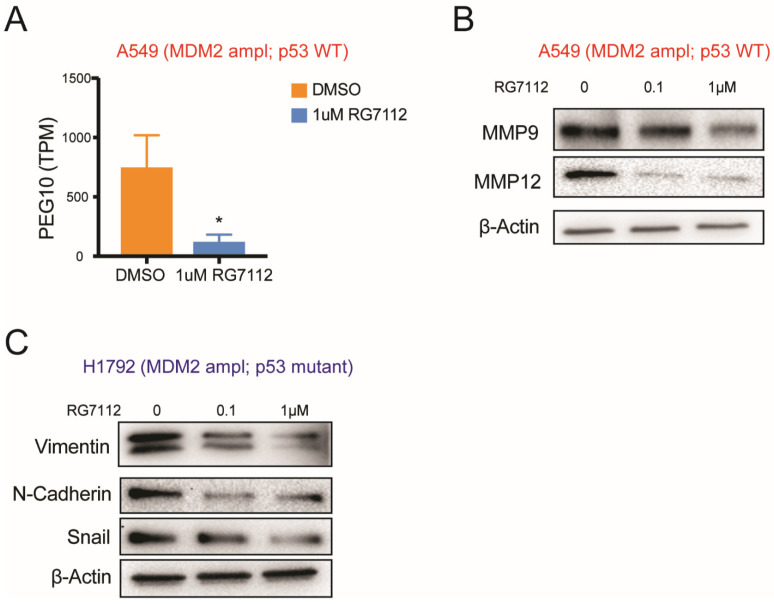
MDM2-targeted therapy suppresses tumor invasiveness by targeting E2F → PEG10 → MMP signaling axis and EMT signaling in p53 wild-type and mutant condition, respectively. (**A**) PEG10 TPM value in A549 (MDM2 ampl; p53 WT) on treatment with DMSO or 1 µM RG7112 from RNA seq data. * *p* < 0.01, n = 3 (**B**) Western blot for MMP9, MMP12 and β-actin in A549 (MDM2 ampl; p53 WT) on treatment with DMSO, 0.1 µM RG7112 or 1 µM RG7112 for 48 h. (**C**) Western blot for Vimentin, N-Cadherin, Snail and β-actin in H1792 (MDM2 ampl; p53 mutant) on treatment with DMSO, 0.1 µM RG7112 or 1 µM RG7112 for 48 h.

E2F has been shown to regulate cell motility and invasion through PEG10 in several tumors [30,31,32]. We observed significant decrease in gene expression of PEG10 in A549 cells treated with RG7112 in our RNA seq data (Figure 6A). PEG10 has been reported to alter tumor cell invasion and metastasis by up-regulating expression of matrix metalloproteinases (MMPs) [30,31,32]. Our Western blot data showed suppression in MMP9 and MMP12 on treatment with RG7112 in MDM2 ampl; p53 WT A549 cells (Figure 6B, Table S8). These data suggested that in MDM2-amplified p53 wild type condition, MDM2-targeted therapy shows tumor-suppressive response by suppressing p53 → E2F → PEG10 → MMP signaling in LUAD.

### 3.7. MDM2 Inhibitor Suppresses Invasiveness in MDM2 Amplified; p53 Mutant Group through Suppressing EMT Signaling

We observed a reduction in migration and invasion in MDM2-amplified cells with p53 mutation on treatment with MDM2 inhibitors (Figure 3 and Figure 4). We hypothesized there must be some p53-independent mechanism responsible for the observed response to drug therapy in MDM2-amplified condition. Our GO analysis identified EMT as top enriched signaling in p53 mutant condition (Figure 5 and Figure 6).

Our Western blot in MDM2-amplified and p53 mutant cell line H1792 validated suppression of EMT markers such as Vimentin, N-Cadherin and Snail on treatment with RG7112 in dose dependent manner (Figure 6C, Table S8). Thus, our data signifies that response to MDM2-targeted therapy in MDM2 amplified; p53 mutant genetic background is through suppressing EMT pathway in LUAD.

## 4. Discussion

MDM2 is frequently amplified and overexpressed in several tumors [33,34,35,36]. It is a classical negative regulator of tumor suppressor gene p53 [37] and therefore, targeting MDM2–p53 interaction has therapeutic potential in many tumor types. Several Phase 1/2/3 clinical trial studies are ongoing using MDM2–p53-inhibitor drugs in patients with several malignancies. Idasanutlin (RG73388), the most advanced derivative of nutlin family, was examined in Phase I clinical trial for acute myeloid leukemia (AML) [38] (Clinicaltrials.gov identifier: NCT01773408) and in Phase III clinical trial for relapsed/refractory AML [39] (ClinicalTrials.gov Identifier: NCT02545283). AMG-232, a selective and high-affinity piperidinone MDM2 inhibitor, is in clinical development for several malignancies [40]. AMG-232 is currently in Phase Ia/Ib clinical trials for metastatic melanoma (ClinicalTrials.gov Identifier: NCT02110355). Lung adenocarcinoma is a highly heterogeneous disease with patients displaying variable genomic and genetic alterations resulting in highly differential tumor biology [41]. This inter-patient heterogeneity is a critical reason for differential response to targeted therapies and thus results in different survival outcomes.

Our SNP array data showed that MDM2 is amplified in 15% of our early stage LUAD patient samples, with increasing rates from AIS/MIA to predominantly invasive subtypes. Our CISH/FISH data validated MDM2 amplification in 29% of patients with LUAD and further underscored a relationship between MDM2 amplification, and overall survival. Both MDM2 amplification and p53 genetic mutation contribute to LUAD biological heterogeneity (Tables S2 and S3). The impact of this highly complex genomic and genetic heterogeneity on patient’s response to MDM2-inhibitor therapy has not been previously investigated in early stage LUAD patients. In this study we uncovered how genomic and genetic heterogeneity influence the response to MDM2-targeted therapy. Our immunohistochemistry data on our tissue microarray from LUAD patient cohort showed a strong correlation of MDM2 CN gain and high protein expression.

To mimic the heterogeneity in LUAD patients, we chose a panel of LUAD cell lines with MDM2 amplification and high expression with or without p53 mutation versus MDM2 diploid and lower expression. We tested effect of MDM2–p53 inhibitor on cell proliferation, cell migration, invasion and wound-healing ability in the panel of LUAD cell lines. Interestingly, we observed a significant decrease in migration, invasion and wound healing in cells with MDM2 amplification and overexpression but not in cells with diploid MDM2, suggesting that response to MDM2-targeted therapy is MDM2 amplification dependent.

The response to MDM2-inhibitor therapy was observed in both p53 wild-type as well as mutant condition in cells with MDM2 amplification. To understand the novel mechanism through which the MDM2-targeted therapy might be influencing tumor progression, we performed RNA seq on A549 (MDM2 amplified; p53 WT) and H1792 (MDM2 amplified; p53 mutant) cells treated with DMSO or RG7112 (MDM2 inhibitor). Our RNA seq analysis revealed unique pathways in the two different p53 genetic backgrounds. In p53 WT condition, MDM2-inhibitor therapy suppresses tumor invasiveness possibly through E2F → PEG10 → MMP pathway, while in the MDM2-amplified and p53 mutant group, MDM2-targeted therapy suppresses EMT signaling that leads to tumor regression. While our study included a limited panel of cell lines for genomic analysis, a follow-up study with a larger panel of cell lines would provide more strength to our findings. Lung adenocarcinoma patients with MDM2 amplification displayed poor survival as compared to those with normal MDM2 CN. Our study suggests that MDM2 amplification/overexpression can be investigated as a clinical biomarker to target lung cancer patients who may benefit from MDM2-targeted therapy.

## 5. Conclusions

In summary, our study uncovers the criteria to select the right target population of LUAD patients that can benefit from MDM2-targeted therapy based on their genomic alteration. Our study shows that only patients with MDM2 CN gain would respond to therapy, thus providing a direction in decision making for their treatment regimen. We reveal that MDM2-inhibitor therapy is effective in MDM2-amplified condition independent of their p53 status; however, the pathway of suppressing tumor progression is unique in both conditions. Our study uncovers that in p53 WT condition MDM2 inhibitor acts through suppressing the novel p53 → E2F → PEG10 pathway, on the other hand in p53 mutant condition it alters EMT signaling leading to reduced tumor invasiveness. Our findings have potential implications in helping clinicians to choose the right patients for therapeutic treatment among the heterogeneous LUAD patients.

## Data Availability

RNA seq data generated in this study is deposited to GEO database with accession code GSE191171.

## Supplementary Materials

The following are available online at www.mdpi.com/article/10.3390/cancers14030708/s1, Figure S1: MDM2 amplification contributes to heterogeneity in LUAD patients, Figure S2: MDM2 copy number amplification is correlated with protein overexpression in LUAD cell lines, Figure S3: Modest effect of MDM2 inhibitor on cell proliferation is MDM2 dependent, Figure S4: RNA seq data for LUAD cell lines, Figure S5: MDM2 inhibitor alters unique signaling and transcription targets in MDM2 amplified; p53 mutant vs. WT background, Table S1: MDM2 FISH/CISH: NY cohort, Table S2: MDM2 and p53 heterogeneity in NY cohort, Table S3: MDM2 and p53 heterogeneity in Broad cohort, Table S4: *p* values for wound healing assay, Table S5: Common and unique downregulated DEGs in A549 and H1792, Table S6: Gene set-enrichment analysis for Hallmark gene sets, C2 curated gene sets and transcription targets using unique downregulated genes in A549, Table S7: Gene set enrichment analysis for Hallmark gene sets, C2 curated gene sets and transcription targets using unique downregulated genes in H1792, Table S8: List of antibodies used in the study, Table S9: Patient’s Clinicopathological information. All the whole western blot figures can be found in the supplementary materials.
