# Supplementary material for: Early-Stage Lung Adenocarcinoma MDM2 Genomic Amplification Predicts Clinical Outcome and Response to Targeted Therapy
Source: Cancers (Basel). 2022 Jan 29;14(3):708. doi: 10.3390/cancers14030708 (PMC8833784; doi:10.3390/cancers14030708)
Supplement: Supplementary file 1 [file cancers-14-00708-s001.zip › Tables S1-8.pdf]

Submit to SI "Tumor heterogeneity"

Research Article

Early-Stage Lung adenocarcinoma MDM2 genomic heterogeneity predicts clinical outcome and response to targeted therapy

Abhilasha Sinha[1,2], Yong Zou[3], Ayushi S. Patel[1,2,4], Seungyeul Yoo[5], Feng Jiang[1,2], Takashi Sato[1,2,6,7], Ranran Kong[1,2,8], Hideo Watanabe[1,2,9], Jun Zhu[2,5,9,10], Pierre P. Massion[3], Alain C. Borczuk[11], Charles A. Powell[1,2,*]

Supplementary Tables

Table S1. MDM2 FISH/CISH: NY cohort

	Total patients; n=111	Percentage
MDM2 aneu+gain	32	29%
MDM2 diploid	79	71%

Table S2. MDM2 and p53 heterogeneity in NY cohort

	MDM2 aneu +gain; n=32	Percentage
p53 WT	22	68%
p53 mut	10	31%

	MDM2 diploid; n=79	Percentage
p53 WT	66	83%
p53 mut	13	16%

Table S3. MDM2 and p53 heterogeneity in Broad cohort

	No of patients	Percentage
Total	183	
MDM2 amplified	21	11%
MDM2 diploid	162	88%

	MDM2 amplified; n=21	Percentage
p53 WT	11	52%
p53 mut	10	48%

	MDM2 diploid; n=162	Percentage
p53 WT	76	47%
p53 mut	86	53%

Table S4. P-values for wound healing assay

	24hrs		72hrs	
Cell lines	DMSO vs 0.1μM RG7112	DMSO vs 1μM RG7112	DMSO vs 0.1μM RG7112	DMSO vs 1μM RG7112
H2009	0.9983	0.8135	0.8503	0.941
H358	0.7171	0.787	0.676	0.7423

Table S5. Common and Unique downregulated DEGs in A549 and H1792

Common n=6	Unique to A549 n=326	Unique to H1792 n=367
POLA2	DBF4	MAD1L1
POLD1	NCAPD2	PLXND1
TCOF1	ANLN	UPF1
CDC20	BRCA1	ITGA3
PLK1	TACC3	CCDC124
HSPA4	DEPDC1	PIGQ
	DEPDC1B	MRC2
	TDP1	PPP5C
	WWC3	ZNF839
	RFC2	PNPLA6
	RAD51	EDC4
	NOP58	AP2S1

	USP13	PHKA2
	CDON	OFD1
	MCM10	CYBA
	KARS	RRP12
	MTHFD2	MSMO1
	ASPM	PITHD1
	KIF26A	SEC61A1
	PRR11	SNRNP40
	POLR1A	RNF4
	SMC1A	CTSA
	HMMR	IARS2
	MCM2	ATP1B3
	GTSE1	CLTCL1
	ADD2	FSTL3
	SPAG5	JMJD6
	RAP1GAP	MBD3
	UBE2T	CPSF1
	FBLN1	PVR
	KIF22	SCARB1
	NDC80	SELENOO
	AURKA	SDHA
	FUS	MRPS34
	BIRC5	MCM6
	WDR76	PLXNA2
	PHGDH	GPATCH1
	CLSPN	XAB2
	CDC45	GTF3C1
	CDC6	NRDC
	CBX5	THOC1
	HNRNPH3	XRCC5
	PRTFDC1	COL16A1
	RANBP1	CAD
	CENPM	POMGNT1
	POLDIP3	RAD54L
	MCM5	ALG9
	CDKN3	SF3B2
	MYBL2	SMOX
	STK4	DDX24
	DIDO1	ARHGAP4
	NOP56	SLC9A1

	FAM83D	EZR
	SYTL4	TBC1D2
	CENPI	ATP5F1D
	DHODH	POLRMT
	CMC2	TSPO
	CEP152	RIN3
	PDGFRL	MTHFD1
	PYCR3	REC8
	MCM4	PLTP
	RNASEH2A	PCIF1
	ASF1B	NINL
	FBL	GMEB2
	SLC1A5	PIGU
	MPP6	APMAP
	H2AFV	TIMP1
	NUDT1	NUP93
	LSM5	VAC14
	RPA3	COG4
	EZH2	COTL1
	EXOSC3	SLC7A5
	NSMCE4A	STUB1
	NPM3	RPAP1
	UBE2S	UBE2W
	RPS6KB1	TSTA3
	C1QBP	MAN2B1
	NSD2	FSD1
	NCAPG	APLP1
	MLEC	TYK2
	MAGOHB	NAPA
	FOXM1	BCAT2
	RAD51AP1	RPL18A
	RFC5	FKBP8
	NUP107	ATP13A1
	TIMELESS	EPHB6
	CDCA3	NSUN5P2
	SRSF3	BUD31
	GMNN	PLOD3
	KIF20A	EDF1
	LMNB1	DNMBP
	BRIX1	RECQL5

	SMC4	DRG2
	ECT2	ATG2A
	UMPS	FOXRED1
	CENPA	CCDC86
	SRSF7	TMEM109
	MSH6	P3H3
	CACYBP	ATP5F1B
	STMN1	KRT18
	NEK2	CHD4
	CENPF	SPSB2
	CTNNAL1	ENO2
	PHF19	SRSF9
	HELLS	MCM3
	TMPO	PPP2R5D
	NCAPH	CUL9
	KIF18A	COL7A1
	HNRNPA2B1	RPS15
	ZWINT	MOGS
	CENPK	ABCB6
	CDK2	SRM
	TUBA1B	MIIP
	HJURP	PLEKHM2
	DEK	ZBTB17
	C17orf53	MRPS15
	C1orf61	P3H1
	SNRPB	ERI3
	MCM8	CTSD
	XRCC3	RPN2
	IGFLR1	CPSF3
	BCL2L12	PTPA
	DLGAP5	ARMH3
	YEATS4	ARAP3
	AUNIP	TBX2
	PKMYT1	RPL21
	FBXL16	ANXA11
	CHTF18	PKN1
	PAICS	TRIR
	PPAT	COQ8B
	ICE2	CHPF
	KNSTRN	PSPN

	PIMREG	SYMPK
	SGO1	ERGIC3
	CEP85	PSMB2
	DNMT1	HECTD3
	DKC1	PRDX5
	NOL11	ASL
	HACL1	TRAP1
	PSMC3IP	UBR4
	TOP2A	EPS15L1
	FAAP24	METTL16
	MRPL35	DGCR8
	RAN	MYO1B
	PCBD2	EIF2AK4
	TOE1	LOXL1
	NASP	FXR2
	CCNB1	DOCK6
	PSRC1	SAFB2
	TMEM106C	MLLT1
	EMP1	TUBGCP2
	CDCA8	ZNF317
	TROAP	HAUS8
	ESPL1	MAP1B
	KDELR2	TRIM47
	ACTL6A	VPS4A
	TRA2B	CTNNBL1
	SMC2	CTIF
	DSCC1	FBH1
	MYC	GNL2
	ARHGEF39	DAGLA
	FOXF2	DHX34
	NUSAP1	ERCC5
	KIF23	CARS2
	KIF20B	DHX9
	BARD1	MRPS9
	HNRNPD	TNS3
	SNRPF	EPRS
	SORD	SAP130
	TLE3	LRRC8A
	PIF1	USP20
	FANCI	TLN1

	TICRR	IFT172
	IMPA2	PREB
	EIF4A3	ENTPD1
	PLK4	ATIC
	NUF2	CERS5
	ANP32E	C12orf10
	DTL	ESYT1
	INTS7	DHX38
	LBR	MBTPS1
	TPRKB	RPS2
	ST6GAL2	SGSM2
	CDCA7	G6PC3
	FANCD2	FKBP10
	CPLX2	PFKL
	TRIM7	SCYL1
	MMS22L	RCN3
	CDCA5	HSPG2
	NCAPG2	RPS8
	GINS4	UCK2
	CEP78	ILF2
	C9orf3	SNAP47
	POLE3	PDIA6
	MKI67	VPS54
	SSRP1	MRPS5
	INCENP	FAHD2B
	CHEK1	HSPD1
	HIRIP3	CCT6A
	SCLT1	NAPRT
	CENPU	CIZ1
	CENPH	PAXX
	SLFN13	NKX6-2
	KCNMA1	PPRC1
	FBXO43	PDCD11
	ATAD2	LIN7C
	BUB1B	APIP
	CSRP1	HYOU1
	IGF2BP1	EML3
	PTMS	FERMT3
	C21orf58	PLCB3
	TOR2A	TMCO3

	LY6K	EEF1AKMT1
	RECQL4	PRSS53
	SRSF2	NBAS
	DBF4B	RAD17
	RACGAP1	TXNDC11
	SPC24	LPCAT1
	SNRNP25	CC2D1B
	TEDC2	TRMT44
	ASRGL1	SUPV3L1
	CIP2A	CCNB2
	TOPBP1	SLC35B2
	RFC4	C2CD2
	H2AFZ	TPRG1L
	HMGB2	TSPAN33
	MAD2L1	ZYX
	PTTG1	CCDC28B
	CDCA7L	GPSM1
	ADCY1	SHKBP1
	RAD21	CRTC2
	AQP3	ALDH16A1
	MELK	LEMD2
	SKA3	POLR3K
	LRR1	PAQR4
	NOLC1	FLYWCH2
	ZNF202	NXF1
	CENPN	LRP5
	WEE1	ATP1A1
	MCM7	INTS1
	SAAL1	SLC4A2
	PCLAF	NOL6
	SGSM1	PMPCA
	SNRPD1	HTRA1
	RRM1	SMPD1
	CDT1	RRAD
	TUBA1C	COG1
	CHAF1A	ANPEP
	SLC3A2	VPS39
	PBK	ACSF2
	ACOX2	SLC27A4
	REEP4	IRGQ

	CKAP2L	ZNF598
	BUB1	SRRM2
	LRRC45	DDB1
	CHRNA5	HEXIM2
	CENPX	CHTF8
	CDK1	ATOH8
	FOS	ATP5ME
	ARL6IP1	SPNS1
	FGG	ASPSCR1
	FGB	FASN
	RRM2	CHD3
	SLFN11	METTL7B
	CKS1B	EXOSC10
	C11orf86	TRAPPC12
	EXO1	LAMB2
	UBE2C	BSG
	CCNE2	MCRIP2
	TOMM5	PPP1CA
	SHMT1	PARP14
	HASPIN	MAP3K11
	LCORL	EHBP1L1
	GEN1	PPP1R14B
	APOLD1	SPTBN2
	PFAS	PRPF8
	AURKB	SEZ6L2
	APOBEC3B	ASPHD1
	TCEAL8	MED16
	SNRPE	SART1
	RFLNB	RPS6KB2
	TRAIP	TPRN
	PTP4A2	RNPEP
	CMSS1	WDR25
	CDCA2	ZDHHC13
	LRRC26	POLE
	ATL3	RPLP2
	SIVA1	PNPLA2
	HIST1H2BL	FLII
	TEDC1	COX14
	KIF18B	TUFM
	KIF24	CYC1

	PTMA	TMEM86B
	SAMD11	ARHGAP45
	FANCA	TRAPPC5
	H2AFX	LDOC1
	HMGB1	SYNM
	XRCC2	COL18A1
	BLM	EP400
	GRK6	ZNF530
	PRIM1	SMTN
	ZNF544	EIF3C
	ARHGAP11A	NOMO2
	HMGN2	AHNAK2
	PRC1	TRIM69
	CENPW	HYAL3
	KIFC1	ZACN
	LGR4	DHRS4L2
	MXD3	EIF4EBP1
	HAUS7	AGRN
	EMP2	LONP1
	DDX12P	ASB13
	TUBB	PLXNB2
	TCF19	MRTFA
	MDC1	FLNA
	ZBTB12	AP2A1
	MRPS17	ANXA6
	RPP21	IGF2R
	PEG10	PCNX3
	HAUS5	SPTAN1
	CENPS-CORT	GPAA1
	SKA1	DDX39B
	FAM72C	GLMP
	NLRP2	SKIV2L
	UHRF1	DHX16
	MYO19	C6orf136
		DCTN1
		RING1
		PBX2
		EHMT2
		NDUFS3
		SLC35F6

		TAX1BP3
		RTL10
		VPS16
		HSPA1A
		TRIM27
		FADS3
		VARS
		PRRC2A
		C19orf24
		ACBD6
		CLIC1
		MSH5
		FLOT1
		ZBTB9
		RPL13AP5
		GET4
		MIF
		ARPC1A
		PI4KA
		PLA2G4B
		GLI4
		CUX1
		RTEL1
		INAFM2
		LOC729218
		GART
		EGLN2
		KMT2B
		TM4SF19-TCTEX1D2
		PI4KAP1
		CNOT3
		IFI27L2
		PSMB3
		ABR
		DEAF1

Table S6. Gene set enrichment analysis for Hallmark gene sets, C2 curated gene sets and transcription targets using unique downregulated genes in A549

Hallmark Geneset				
Gene Set Name [# Genes (K)]	Description	# Genes in Overlap (k)	p-value	FDR q-value
HALLMARK_E2F_TARGETS [200]	Genes encoding cell cycle related targets of E2F transcription factors.	84	5.44 e-124	2.72E-122
HALLMARK_G2M_CHECKPOINT [200]	Genes involved in the G2/M checkpoint, as in progression through the cell division cycle.	64	1.23 e-84	3.08E-83
HALLMARK_MYC_TARGETS_V1 [200]	A subgroup of genes regulated by MYC - version 1 (v1).	27	6.1 e-25	1.02E-23
HALLMARK_MITOTIC_SPINDLE [199]	Genes important for mitotic spindle assembly.	23	7.42 e-20	9.27E-19
HALLMARK_SPERMATOGENESIS [135]	Genes up-regulated during production of male gametes (sperm), as in spermatogenesis.	10	1.68 e-7	1.68E-06
HALLMARK_MYC_TARGETS_V2 [58]	A subgroup of genes regulated by MYC - version 2 (v2).	7	4.48 e-7	3.21E-06
HALLMARK_DNA_REPAIR [150]	Genes involved in DNA repair.	10	4.49 e-7	3.21E-06
HALLMARK_ESTROGEN_RESPONSE_LATE [200]	Genes defining late response to estrogen.	11	8.17 e-7	5.11E-06
HALLMARK_UNFOLDED_PROTEIN_RESPONSE [113]	Genes up-regulated during unfolded protein response, a cellular stress response related to the endoplasmic reticulum.	8	4.11 e-6	2.29E-05
HALLMARK_MTORC1_SIGNALING [200]	Genes up-regulated through activation of mTORC1 complex.	10	6.04 e-6	3.02E-05
**C2 curated geneset**				

Gene Set Name [# Genes (K)]	Description	# Genes in Overlap (k)	p-value	FDR q-value
FISCHER_DREAM_TARGETS [968]	Target genes of the DREAM complex.	228	6.71 e-298	4.22E-294
GOBERT_OLIGODENDROCYTE_DIFFERENTIATION_UP [571]	Genes up-regulated during later stage of differentiation of Oli-Neu cells (oligodendroglial precursor) in response to PD174265 [PubChem=4709].	168	4.77 e-227	1.50E-223
DUTERTRE_ESTRADIOL_RESPONSE_24HR_UP [323]	Genes up-regulated in MCF7 cells (breast cancer) at 24 h of estradiol [PubChem=5757] treatment.	119	7.5 e-170	1.57E-166
MARSON_BOUND_BY_E2F4_UNSTIMULATED [692]	Genes with promoters bound by E2F4 [GeneID=1874] in unstimulated hybridoma cells.	145	8.38 e-169	1.32E-165
MEBARKI_HCC_PROGENITOR_FZD8CRD_UP [586]	Transcriptome of human HepaRG hepatocellular carcinoma liver progenitors in responses to a WNT3A-enriched microenvironment and dissection of pathways dependent on _-catenin and/or blocked by the SFRP-like Wnt inhibitor FZD8_CRD.	137	8.03 e-166	1.01E-162
KINSEY_TARGETS_OF_EWSR1_FLII_FUSION_UP [1290]	Genes up-regulated in TC71 and EWS502 cells (Ewing's sarcoma) by EWSR1-FLI1 [GeneID=2130;2314] as inferred from RNAi knockdown of this fusion protein.	166	9.42 e-159	9.87E-156

KOBAYASHI_EGFR_SIGNALING_24HR_DN [252]	Genes down-regulated in H1975 cells (non-small cell lung cancer, NSCLC) resistant to gefitinib [PubChem=123631] after treatment with EGFR inhibitor CL-387785 [PubChem=2776] for 24h.	98	2.54 e-141	2.28E-138
NUYTTEN_EZH2_TARGETS_DN [1020]	Genes down-regulated in PC3 cells (prostate cancer) after knockdown of EZH2 [GeneID=2146] by RNAi.	144	4.44 e-141	3.49E-138
FLORIO_NEOCORTEX_BASAL_RADIAL_GLIA_DN [190]	Genes down-regulated in basal radial glia (bRG) relative to apical radial glia (aRG), and up-regulated in both aRG and bRG relative to neurons.	88	5.91 e-135	4.13E-132
DODD_NASOPHARYNGEAL_CARCINOMA_DN [1408]	Genes down-regulated in nasopharyngeal carcinoma (NPC) compared to the normal tissue.	152	1.2 e-131	7.57E-129
**Transcription targets geneset**				
Gene Set Name [# Genes (K)]	Description	# Genes in Overlap (k)	p-value	FDR q-value
HSD17B8_TARGET_GENES [659]	Genes containing one or more binding sites for UniProt:Q92506 (HSD17B8) in their promoter regions (TSS -1000,+100 bp) as identified by	134	6.72 e-153	7.62E-150

	GTRD version 20.06 ChIP-seq harmonization.			
E2F5_TARGET_GENES [1273]	Genes containing one or more binding sites for UniProt:Q15329 (E2F5) in their promoter regions (TSS -1000,+100 bp) as identified by GTRD version 20.06 ChIP-seq harmonization.	79	2.12 e-46	1.20E-43
E2F_Q6 [236]	Genes having at least one occurence of the transcription factor binding site V$E2F_Q6 (v7.4 TRANSFAC) in the regions spanning up to 4 kb around their transcription starting sites.	39	3.76 e-39	1.42E-36
E2F_Q4 [238]	Genes having at least one occurence of the transcription factor binding site V$E2F_Q4 (v7.4 TRANSFAC) in the regions spanning up to 4 kb around their transcription starting sites.	39	5.3 e-39	1.50E-36
BARX2_TARGET_GENES [17 23]	Genes containing one or more binding sites for UniProt:Q9UMQ3 (BARX2) in their promoter regions (TSS -1000,+100 bp) as identified by GTRD version 20.06 ChIP-seq harmonization.	77	3.18 e-35	7.20E-33
E2F1DP1_01 [238]	Genes having at least one occurence of the transcription factor binding site V$E2F1DP1_01	36	9.38 e-35	1.18E-32

	(v7.4 TRANSFAC) in the regions spanning up to 4 kb around their transcription starting sites.			
E2F1DP2_01 [238]	Genes having at least one occurence of the transcription factor binding site V$E2F1DP2_01 (v7.4 TRANSFAC) in the regions spanning up to 4 kb around their transcription starting sites.	36	9.38 e-35	1.18E-32
E2F4DP2_01 [238]	Genes having at least one occurence of the transcription factor binding site V$E2F4DP2_01 (v7.4 TRANSFAC) in the regions spanning up to 4 kb around their transcription starting sites.	36	9.38 e-35	1.18E-32
E2F_02 [238]	Genes having at least one occurence of the transcription factor binding site V$E2F_02 (v7.4 TRANSFAC) in the regions spanning up to 4 kb around their transcription starting sites.	36	9.38 e-35	1.18E-32
E2F_Q6_01 [242]	Genes having at least one occurence of the transcription factor binding site V$E2F_Q6_01 (v7.4 TRANSFAC) in the regions spanning up to 4 kb around their transcription starting sites.	36	1.74 e-34	1.98E-32

Table S7. Gene set enrichment analysis for Hallmark gene sets, C2 curated gene sets and transcription targets using unique downregulated genes in H1792

**Hallmark Geneset**				
Gene Set Name [# Genes (K)]	Description	# Genes in Overlap (k)	p-value	FDR q-value
HALLMARK_EPITHELIAL_MESENCHYMAL_TRANSINSITION [200]	Genes defining epithelial-mesenchymal transition, as in wound healing, fibrosis and metastasis.	13	4.43 e-8	2.21E-06
HALLMARK_UNFOLDED_PROTEIN_RESPONSE [113]	Genes up-regulated during unfolded protein response, a cellular stress response related to the endoplasmic reticulum.	9	9.89 e-7	2.47E-05
HALLMARK_MYC_TARGETS_V1 [200]	A subgroup of genes regulated by MYC - version 1 (v1).	11	2.5 e-6	4.17E-05
HALLMARK_MITOTIC_SPINDLE [199]	Genes important for mitotic spindle assembly.	9	9.41 e-5	9.78E-04
HALLMARK_OXIDATIVE_PHOSPHORYLATION [200]	Genes encoding proteins involved in oxidative phosphorylation.	9	9.78 e-5	9.78E-04
HALLMARK_MYC_TARGETS_V2 [58]	A subgroup of genes regulated by MYC - version 2 (v2).	5	1.85 e-4	1.54E-03
HALLMARK_PROTEIN_SECRETION [96]	Genes involved in protein secretion pathway.	6	2.51 e-4	1.79E-03
HALLMARK_G2M_CHECKPOINT [200]	Genes involved in the G2/M checkpoint, as in progression through the cell division cycle.	8	5.21 e-4	2.89E-03
HALLMARK_GLYCOLYSIS [200]	Genes encoding proteins involved in glycolysis and gluconeogenesis.	8	5.21 e-4	2.89E-03
HALLMARK_ADIPOGENESIS [200]	Genes up-regulated during adipocyte differentiation (adipogenesis).	7	2.46 e-3	1.23E-02
**C2 curated geneset**				

Gene Set Name [# Genes (K)]	Description	# Genes in Overlap (k)	p-value	FDR q-value
DEBIASI_APOPTOSIS_BY_REOVIRUS_INFECTIOTION_DN [276]	Genes down-regulated in HEK293 cells (embryonic kidney) at 6 h, 12 h or 24 h after infection with reovirus strain T3A (known as a strong inducer of apoptosis).	33	7.4 e-27	4.65E-23
MILI_PSEUDOPODIA_HAPTOTAXIS_DN [672]	Transcripts depleted from pseudopodia of NIH/3T3 cells (fibroblast) in response to haptotactic migratory stimulus by fibronectin, FN1 [GeneID=2335].	44	1.57 e-24	4.92E-21
KIM_ALL_DISORDERS_OLIGODENDROCYTE_NUMBER_CORR_UP [754]	Genes whose expression was significantly and positively correlated with the number of perineuronal oligodendrocytes in the layer III of BA9 brain region.	41	6 e-20	1.26E-16
WELCSH_BRCA1_TARGETS_DN [141]	Down-regulated by induction of exogenous BRCA1 in EcR-293 cells	21	1.53 e-19	2.40E-16
BLALOCK_ALZHEIMERS_DISEASE_UP [1673]	Genes up-regulated in brain from patients with Alzheimer's disease.	58	1.89 e-18	2.38E-15
REACTOME_METABOLISM_OF_RNA [672]	Metabolism of RNA	37	2.87 e-18	3.01E-15
MILI_PSEUDOPODIA_CHEMOTAXIS_DN [426]	Transcripts depleted in pseudopodia of NIH/3T3 cells (fibroblast) in response to the chemotactic migration stimulus by lysophosphatidic acid (LPA) [PubChem=3988].	28	5.07 e-16	4.55E-13
LOPEZ_MBD_TARGETS [954]	Genes up-regulated in HeLa cells (cervical cancer) after simultaneus knockdown of all three MBD (methyl-CpG	40	1.27 e-15	1.00E-12

	binding domain) proteins MeCP2, MBD1 and MBD2 [GeneID=4204;4152;8932] by RNAi.			
SPIELMAN_LYMPHOBLAST_EUROPEAN_VS_ASIAN_UP [485]	Genes up-regulated in lymphoblastoid cells from the European population compared to those from the Asian population.	29	1.77 e-15	1.24E-12
CAIRO_HEPATOBLASTOMA_CLASSES_UP [612]	Genes up-regulated in robust Cluster 2 (rC2) of hepatoblastoma samples compared to those in the robust Cluster 1 (rC1).	32	2.58 e-15	1.62E-12
**Transcription targets geneset**				
Gene Set Name [# Genes (K)]	Description	# Genes in Overlap (k)	p-value	FDR q-value
FXR1_TARGET_GENES [1011]	Genes containing one or more binding sites for UniProt:P51114 (FXR1) in their promoter regions (TSS -1000,+100 bp) as identified by GTRD version 20.06 ChIP-seq harmonization.	48	9.09 e-21	1.03E-17
JDP2_TARGET_GENES [1891]	Genes containing one or more binding sites for UniProt:Q8WYK2 (JDP2) in their promoter regions (TSS -1000,+100 bp) as identified by GTRD version 20.06 ChIP-seq harmonization.	62	1.61 e-18	9.10E-16
CEBPZ_TARGET_GENES [1520]	Genes containing one or more binding sites for UniProt:Q03701 (CEBPZ) in their promoter regions (TSS -1000,+100 bp) as identified by GTRD version 20.06 ChIP-seq harmonization.	55	2.49 e-18	9.40E-16

NUP153_TARGET_GENES [1634]	Genes containing one or more binding sites for UniProt:P49790 (NUP153) in their promoter regions (TSS -1000,+100 bp) as identified by GTRD version 20.06 ChIP-seq harmonization.	56	1.32 e-17	3.74E-15
SFMBT1_TARGET_GENES [1661]	Genes containing one or more binding sites for UniProt:Q9UHJ3 (SFMBT1) in their promoter regions (TSS -1000,+100 bp) as identified by GTRD version 20.06 ChIP-seq harmonization.	56	2.71 e-17	6.15E-15
SETD7_TARGET_GENES [991]	Genes containing one or more binding sites for UniProt:Q8WTS6 (SETD7) in their promoter regions (TSS -1000,+100 bp) as identified by GTRD version 20.06 ChIP-seq harmonization.	42	1.61 e-16	3.04E-14
KDM7A_TARGET_GENES [1840]	Genes containing one or more binding sites for UniProt:Q6ZMT4 (KDM7A) in their promoter regions (TSS -1000,+100 bp) as identified by GTRD version 20.06 ChIP-seq harmonization.	56	2.24 e-15	3.63E-13
ZNF592_TARGET_GENES [1890]	Genes containing one or more binding sites for UniProt:Q92610 (ZNF592) in their promoter regions (TSS -1000,+100 bp) as identified by GTRD version 20.06 ChIP-seq harmonization.	56	6.93 e-15	9.81E-13
THAP1_TARGET_GENES [1985]	Genes containing one or more binding sites for UniProt:Q9NVV9 (THAP1) in their promoter regions (TSS -	57	1.45 e-14	1.83E-12

	1000,+100 bp) as identified by GTRD version 20.06 ChIP-seq harmonization.			
ZSCAN30_TARGET_GENES [1756]	Genes containing one or more binding sites for UniProt:Q86W11 (ZSCAN30) in their promoter regions (TSS -1000,+100 bp) as identified by GTRD version 20.06 ChIP-seq harmonization.	53	1.93 e-14	2.19E-12

Table S8. List of antibodies used in the study.

Antibody	Source	Identifier
MDM2 (WB)	Abcam	ab16895
P53	Abcam	ab1101
MDM2 (IHC)	Calbiochem	OP46
N-Cadherin	Cell Signaling Technology	13116
Vimentin	Cell Signaling Technology	5741
Snail	Cell Signaling Technology	3879
MMP9	Cell Signaling Technology	13667
MMP12	Abcam	ab52897
β-Actin	Sigma	A5316
Vinculin	Sigma	V4505

Table S9. Patient's Clinicopathological information

originalorder	Stage	Stage	Age	Gender	Histology
1.0	T1A N0	1a	68	M	SOL
2.0	T2A N0	1b	67	F	AC
3.0	T2A N0	1b	59	F	AC
4.0	T1A N0	1a	59	F	MIA

5.0	T1A N0	1a	81	M	MIA
6.0	T1A N0	1a	62	F	MIA
7.0	T2A N0	1b	73	M	AC
8.0	T2A N0	1b	59	F	AC
9.0	T1A N0	1a	75	M	PAP
10.0	T2A N0	1b	78	M	AC
11.0	T2a N0	1b	73	M	AC
12.0	T2A N0	1b	51	M	AC
13.0	T1B N1	2a	72	M	AC
14.0	1a	1a	72	M	AIS
15.0	T1A N0	1a	64	M	MIA
16.0	T1A N0	1a	76	F	MIA
17.0	T1A N0	1a	74	F	MIA
18.0	T2A NX	1b	72	M	AC
19.0	T1A N0	1a	68	F	AC
20.0	T2A N0	1b	90	F	AC
21.0	T1A N0	1a	81	F	AC
22.0	T1A N0	1a	81	F	AIS
23.0	T1B N0	1a	56	F	MIA
24.0	1b	1b	74	F	MIA
25.0	T1A N0	1a	78	M	MIA
26.0	T1A N0	1a	50	F	MIA
27.0	T2A N0	1b	71	F	AIS
28.0	T1B N0	1a	59	M	PAP
29.0	T2A N0	1b	82	F	SOL
30.0	T1B N0	1a	73	F	AC
31.0	T3 N2	3a	74	F	MP
32.0	t2a n0	1b	76	F	MIA
33.0	T1A N0	1a	62	F	AIS
34.0	T1A N0	1a	59	F	AIS
35.0	t1a nx	1a	68	M	MIA
36.0	T1B N0	1a	63	M	AC
37.0	T1B N0	1a	70	M	AC
38.0	T1B N0	1a	65	F	AC
39.0	T1A N0	1a	42	F	LPA
40.0	T1A N0	1a	56	F	MIA
41.0	1b	1b	55	M	MIA
42.0	T1A N0	1a	70	F	MIA
43.0	T1A N0	1a	67	F	MIA
44.0	T1B N0	1a	66	M	AC
45.0	ypT1B N2	3a	72	F	AC
46.0	T1a N0	1a	74	M	AIS
47.0	T2B N2	3a	77	F	MUC

48.0	T1A N0	1a	71	F	AIS
49.0	T1A N0	1a	77	F	MIA
50.0	T2A N0	1b	59	F	MIA
51.0	T1A N0	1a	44	F	AIS
52.0	T1A N0	1a	69	F	AC
53.0	T1A N0	1a	70	M	MP
54.0	T1B N0	1a	64	F	SOL
55.0	T2A N2	3a	65	M	MP
56.0	T1A N0	1a	82	F	AIS
57.0	t1a nmx	1a	90	M	MIA
58.0	1a	1a	76	M	AIS
59.0	T1A N0	1a	53	F	AIS
60.0	T1a NX	1a	82	F	AC
61.0	T1B N0	1a	63	M	MIA
62.0	T1A N0	1a	78	M	MIA
63.0	T1A N1	2a	71	M	PAP
64.0	t1a n0	1a	77	F	AC
65.0	T1A N0	1a	72	F	MIA
66.0	T1A NX	1a	63	F	AC
67.0	T1A NX	1a	63	F	AIS
68.0	1a	1a	54	F	AIS
69.0	T1A N0	1a	73	M	SOL
70.0	T1A N0	1a	57	F	SOL
71.0	T2A N0	1b	59	M	AC
72.0	T1A N0	1a	41	F	MP
73.0	T1A N0	1a	68	F	AIS
74.0	T1A NX	1a	61	F	AIS
75.0	T1A NX	1a	67	F	MIA
76.0	T1A N0	1a	65	F	MIA
77.0	T1B N0	1a	70	M	MUC
78.0	T2A N2	3a	74	F	MP
79.0	T2A N0	1b	80	F	SOL
80.0	T1A N0	1a	67	F	PAP
81.0	T1A NX	1a	15	F	AIS
82.0	T1A Nx	1a	74	M	AIS
83.0	T1A N0	1a	73	F	MIA
84.0	1a	1a	58	F	MIA
85.0	T2A N0	1b	71	M	MUC
86.0	T1A N0	1a	50	F	PAP
87.0	T1A N0	1a	61	F	AC
88.0	T2A N1	2a	67	F	SOL
89.0	T1A N0	1a	64	F	AIS
90.0	T1A NX	1a	75	M	AIS

91.0	T1A N0	1a	77	F	AIS
92.0	T1A N0	1a	69	M	MIA
93.0	1a	1a	73	M	AIS
94.0	T1A N0	1a	73	M	PAP
95.0	T2A N0	1b	70	F	MIA
96.0	T1A N0	1a	62	F	AIS
97.0	T4 N0	3a	67	F	AC
98.0	T2A N0	1b	76	F	AC
99.0	1a	1a	76	F	AIS
100.0	T1A N0	1a	52	M	SOL
101.0	T2A N1	2a	73	M	SOL
102.0	T1A N0	1a	56	M	AIS
104.0	T1A Nx	1a	73	F	AIS
104.5	T1A Nx	1a	73	f	
105.0	T1A N0	1a	49	F	AIS
106.0	T3 N1	3a	77	M	Adsq
107.0	T2A N2	3a	82	M	Adsq
108.0	T1A N0	1a	68	F	PAP
109.0	t1a n0	1a	71	M	AC
110.0	T1B N0	1a	52	F	AC
111.0	T1A N0	1a	68	F	AIS
112.0	T1A N0	1a	67	F	MIA
113.0	T1A N0	1a	49	F	MIA
114.0	T1A N0	1a	60	M	MIA
115.0	T1A N0	1a	65	F	MIA
116.0	T2A N0	1b	57	F	AC
117.0	ypT1B N2	3a	78	M	MP
118.0	T2A N0	1b	57	F	SOL
119.0	t1b n0	1a	77	F	MP
120.0	T1A NX	1a	80	F	MP
121.0	T2A N0	1b	63	M	PAP
122.0	T2A N0	1b	65	F	SOL
123.0	T1A N0	1a	75	M	AC
124.0	T1A N0	1a	63	F	MP
125.0	T2A N2	3a	77	F	AC
126.0	T1A N0	1a	65	F	AC
127.0	T2A N0	1b	82	F	PAP
128.0	T2B N0	2a	80	F	PAP
129.0	T2A N1	2a	66	F	AC
130.0	T1A N0	1a	83	F	AC
132.0		1	68	F	AIS
133.0	t2an2	3a	62	F	SOL
134.0	t1an0	1a	74	F	AC

135.0	t1an0	1a	66	F	AC
136.0	t2an1	2a	61	M	PAP
137.0	t1bn2	3a	59	F	PAP
138.0	t2an0	1b	63	F	SOL
139.0	t2an0	1b	87	F	PAP
140.0	t2an0	1b	80	F	AC
141.0	t1bn0	1a	75	F	AC
142.0	t1bn0	1a	78	F	PAP
143.0	t1bn0	1a	78	F	PAP
144.0			77	F	AC
145.0	t1an0	1a	80	F	AC
147.0	t1an0	1a	77	F	AC
149.0	t1an0	1a	67	F	AC
150.0	1a	1a	67	F	AC
151.0	1b	1b	79	F	AC
152.0	1A	1a	66	F	AC
153.0	3a	3a	60	M	AC
154.0	1a	1a	71	F	MIA
155.0	1b	1b	58	M	SOL
156.0	1a	1a	53	F	ADSQ
157.0	1a	1a	67	M	LPA
158.0	1b	1b	76	F	MIA
159.0	3a	3a	80	F	PAP
160.0	1a	1a	68	M	AC
161.0	1a	1a	80	M	AC
162.0	1b	1b	75	M	AC
163.0	1a	1a	70	F	AC
164.0	1b	1b	78	M	MIA
165.0	1a	1a	73	M	SOL
166.0	3a	3a	47	M	AC
167.0	1a	1a	60	M	AC
168.0	1a	1a	58	F	AC
169.0	3a	3a	72	M	PAP
170.0	1b	1b	70	M	PAP
171.0	1a	1a	76	M	MIA
172.0	1b	1b	57	F	PAP
173.0	1a	1a	77	F	AC
174.0	1b	1b	63	M	AC
175.0	1b	1b	67	M	SOL
176.0	1a	1a	62	F	AC
177.0	1a	1a	75	F	AC
178.0	2b	2b	59	M	AC
179.0	3a	3a	48	M	SOL

180.0	1a	1a	69	F	AC
181.0	1b	1b	79	M	AC
182.0	1a	1a	56	F	MIA
183.0	1b	1b	70	F	MIA
184.0	1a	1a	86	F	MIA
185.0	3a	3a	52	M	AC
186.0	3b	3b	72	F	AC
187.0	1a	1a	75	F	AC
188.0	3a	3a	75	M	AC
189.0	1b	1b	72	F	AC
190.0	1a	1a	74	F	AC
191.0	1b	1b	57	M	AC
192.0	1A	1A	56	M	AC
193.0	1a	1a	78	M	MIA
194.0	2b	2b	70	F	ADSQ
195.0	2a	2a	71	M	MP
196.0	1b	1b	79	F	MP
197.0	1b	1b	55	M	MIA
198.0	1b ©	1b	79	F	PAP
199.0	2a	2a	69	M	AC
200.0	1b	1b	66	F	PAP
201.0	1a	1a	59	M	PAP
202.0	1a ©	1a	60	F	MIA
203.0	1a	1a	69	F	MIA
204.0	1b or 3b	1b	83	F	AC
205.0	1a	1a	70	F	AC
206.0	2a	2a	61	M	SOL
207.0	2b	2b	66	M	AC
208.0	1a	1a	75	F	MIA
209.0	3a	3a	78	M	SOL
210.0	1B	1B	#NULL!	F	AC
211.0	4	4	53	M	MUC
212.0	1a	1a	54	M	AIS
213.0	3a	3a	72	M	SOL
214.0	1b	1b	55	F	SOL
215.0	1a	1a	66	M	PAP
216.0	t2an0	1b	77	F	PAP
217.0	1b	1b	64	M	AC
218.0	1b	1b	62	M	AC
219.0	1b	1b	72	F	MIA
220.0	2b	2b	63	F	AC
221.0	1a	1a	#NULL!	F	MIA
222.0			#NULL!	F	SOL

223.0	1a	1a	63	F	MIA
224.0	1b	1b	47	M	SOL
225.0	1a	1a	65	M	SOL
226.0	1a	1a	68	M	SOL
227.0			#NULL!	F	SOL
228.0	1b	1b	81	M	AC
229.0	1a	1a	64	M	ADSQ
230.0	1b	1b	56	F	SOL
231.0	1b	1b	59	F	AC
232.0	1a	1a	#NULL!	F	AC
233.0	1b	1b	58	F	MIA
234.0	1a	1a	68	F	PAP
235.0	1a	1a	55	F	AC
236.0	1b	1b	72	F	LPA
237.0	1a	1a	80	F	MIA
238.0	3a	3a	54	M	PAP
239.0	2b	2b	71	F	AC
240.0	3a	3a	67	F	AC
241.0	2a	2a	52	F	PAP
242.0	3a	3a	67	F	SOL
243.0	t2an0	1b	69	F	AC
244.0	1a	1a	69	F	MP
245.0	1b	1b	71	M	LPA
246.0	1a	1a	70	F	SOL
247.0	1a	1a	64	F	PAP
248.0	1a	1a	63	F	MIA
249.0	3a or 3b	3a	61	F	AC
250.0	1a	1a	60	F	MIA
251.0	1a	1a	56	F	AC
252.0	1a	1a	62	F	AIS
253.0	1a	1a	60	F	AC
254.0	2a	2a	71	M	AC
255.0	1b	1b	74	F	PAP
256.0	1a	1a	66	F	MUC
257.0	1b	1b	73	M	AC
258.0	3a	3a	69	M	SOL
259.0			#NULL!	M	MP
260.0	3a	3a	#NULL!	M	AC
261.0	2a	2a	62	F	AC
262.0	1a	1a	70	F	AC
263.0	3a	3a	73	F	PAP
264.0	2a	2a	55	M	AC
265.0	1a	1a	50	F	SOL

266.0	2a	2a	73	M	PAP
267.0	1a	1a	62	F	AIS
268.0	1a	1a	70	F	MIA
269.0	1a	1a	62	F	SOL
270.0	1a	1a	73	F	AC
271.0	1a	1a	81	M	PAP
272.0	1a	1a	66	M	AC
273.0	t1bn1	2a	60	F	SOL
274.0	1b	1b	66	F	LPA
275.0	1a	1a	73	M	MP
276.0	2B	2B	69	F	LPA
277.0	1b	1b	60	F	AC
278.0	3A	3A	53	F	AC
279.0	t2an0	1b	73	F	SOL
280.0	2b	2b	76	F	ADSQ
281.0	4	4	55	M	AC
282.0	1b	1b	71	F	AC
283.0	2a	2a	54	M	MUC
284.0			76	M	SOL
285.0	1A	1A	72	M	AIS
286.0	1b	1b	70	F	SOL
287.0	1b	1b	74	F	MUC
288.0	1a	1a	67	F	MIA
289.0	2b	2b	77	F	AC
290.0	1a	1a	48	F	MUC
291.0	1b	1b	54	F	AC
292.0	1b	1b	58	F	SOL
293.0	1a	1a	46	F	SOL
294.0	1a	1a	61	F	SOL
295.0	2b	2b	82	F	PAP
296.0	1a	1a	68	F	AC
297.0	1a	1a	76	F	AC
298.0	1a	1a	65	F	MIA
299.0	1b	1b	76	F	AC
300.0	1a	1a	71	M	MIA
301.0	1a	1a	66	F	AC
302.0	t2an0	1b	82	M	AC
303.0	2b	2b	75	M	AC
304.0	1a	1a	87	M	AC
305.0	1b	1b	74	M	MUC
306.0	3a	3a	48	M	MP
307.0	3a	3a	45	F	SOL
308.0	2a	2a	53	F	PAP

309.0	1A	1A	66	F	AC
310.0	1b	1b	59	F	MIA
311.0	1a	1a	60	F	AIS
312.0	3a	3a	69	M	SOL
313.0	1A	1A	69	F	AC
314.0	1a	1a	58	F	AC
315.0	3a	3a	61	M	MUC
316.0	1b	1b	61	M	MUC
317.0	1a	1a	49	F	LPA
318.0	1A	1A	63	F	AIS
319.0	1a	1a	64	M	AIS
320.0	1b (stage iv bac in other lobe)	1b	72	M	PAP
321.0	iv (2 nodules)	4	62	F	SOL
322.0	1a	1a	74	F	AC
323.0	1a	1a	68	F	PAP
324.0	1a	1a	61	M	PAP
325.0	2b	2b	74	F	PAP
326.0	1a	1a	54	M	LPA
327.0	2a	2a	80	F	AC
328.0	1b	1b	76	F	MIA
329.0	1b	1b	75	M	AC
330.0			61	F	SOL
331.0	1b	1b	76	F	AC
332.0	1a	1a	76	F	AIS
333.0	1b	1b	76	M	SOL
334.0	1a	1a	71	F	AIS
336.0	1a	1a	62	F	MP
337.0	1a	1a	44	F	AIS
338.0			56	F	AC
339.0	t2an0	1b	87	F	AC
340.0	1a	1a	81	F	MIA
341.0	1a	1a	75	F	LPA
342.0	t3n0	2b	60	F	AC
343.0	1a	1a	86	F	LPA
344.0	1a	1a	57	F	MIA
345.0	T1B N0	1a	79	F	MP
346.0	T3 N2	3a	80	M	SARC
347.0	2A	2a	64	M	LCC
348.0	T1A N0	1a	76	F	AC
349.0	T3 (M) N0	2b	83	F	AC
350.0	T2A N0	1b	73	F	LPA
351.0	T1A N0	1a	59	F	MIA

352.0	2b	2b	56	M	LCC
353.0	t2a n2	3a	70	F	AC
354.0	2a	2a	71	M	SQ
355.0	2b	2b	68	F	LCC
356.0	T1A N0	1a	50	F	MIA
357.0	YPT3N1	3a	59	M	SOL
358.0	T2A N0	1b	73	M	AC
359.0	2a	2a	80	M	LCC
360.0	T1A N0	1a	77	F	LPA
361.0	1a	1a	58	F	LCC
362.0	T2A N0	1b	69	M	MUC
363.0	1b	1b	55	F	LCC
364.0	T1B N0	1a	59	F	ADSQ
365.0	T2A N0	1b	71	F	AC
366.0	2a	2a	62	F	LCC
367.0	1b	1b	62	M	SQ
368.0	T1A N0	1a	66	M	SOL
369.0	1b	1b	74	M	SARC
370.0	1b	1b	81	M	LCC
371.0	t1bn0	1a	64	M	CARCINOID
372.0	1a	1a	86	M	SQ
373.0	t3n0	2b	65	F	LCC
374.0	1a	1a	71	M	SQ
375.0	T1B N1	2a	58	M	SOL
376.0	T2A N0	1b	72	F	AC
377.0	T1A N0	1a	72	F	MIA
378.0	T2A N0	1b	78	M	PAP
379.0	T1B N0	1a	78	F	SOL
380.0	T1B N0	1a	53	F	AC
381.0	1a	1a	83	F	SQ
382.0	t2an1	2a	68	M	LCC
383.0	T3 N1	3a	85	F	SOL
384.0	T3 N1	3a	85	F	SOL
385.0	T1A N0	1a	65	F	SOL
386.0	t2an1	2a	78	M	LCC
387.0	2a	2a	51	M	LCC
388.0	2a	2a	55	F	LCC
389.0	t2an0	1b	75	M	LCC
390.0	t3n0	2b	43	M	SARC
391.0	T2A N0	1b	72	M	PAP
392.0	T1A N0	1a	58	F	LPA
393.0	T1A N0	1a	74	M	PAP
394.0	1b	1b	60	M	LCNEC

395.0	T3 N0	2b	80	M	SOL
396.0	T3 (M) N0	2b	77	F	AC
397.0	T2A N0	1b	76	M	AC
398.0	2b	2b	75	F	SARC
399.0	T1A N0	1a	70	F	AC
400.0	2A	2a	81	M	SQ
401.0	T1A N0	1a	69	M	MUC
402.0	T1B N0	1a	79	F	LPA
403.0	2a	2a	68	F	LCC
404.0	T2A N0	1b	67	F	AC
405.0			62	M	SQ
406.0	T2A N0	1b	64	M	AC
407.0	4	4	77	F	SQ
408.0	T3 N2	3a	71	M	ADSQ
409.0	T2A N0	1b	77	F	MUC
410.0	2a	2a	71	M	SQ
411.0	ypT1aN0 yp1a	1a	45	M	MP
412.0	T1A N0	1a	72	F	PAP
413.0	1a	1a	68	M	SQ
414.0	1a	1a	82	F	SARC
415.0	T2A N0	1b	66	M	PAP
416.0	T1A N0	1a	66	M	MP
417.0	T1A N0	1a	67	M	MP
418.0	1b	1b	76	F	SQ
419.0	T2B N0	2a	75	F	MUC
420.0	1a	1a	73	M	SQ
421.0	1a	1a	53	M	SQ
422.0	T1A N0	1a	63	F	MP
423.0	T2A N1	2a	48	F	AC
424.0	T2A N2	3a	77	F	AC
425.0	T2A N2	3a	82	M	MP
426.0	T1A NX	1a	90	F	LPA
427.0	T4 N0	3a	67	F	AC
428.0	T4 N0	3a	67	F	PAP
429.0	T1A N0	1a	65	F	LPA
430.0	1b	1b	66	F	SARC
431.0	T1A N0	1a	70	F	AC
432.0	T1A NX	1a	56	F	AC
433.0	REP		72	M	AC
434.0	REP		72	M	SQ
435.0	T1A N0	1a	79	M	PAP
436.0	?2b or more	2b	73	M	AC
437.0	T2A N1	2a	66	F	AC

438.0	1b	1b	59	M	SQ
439.0	T1A N0	1a	83	F	AC
440.0	T1B N0	1a	77	F	MUC
441.0	1b	1b	82	M	SQ
442.0	1a	1a	68	F	SQ
443.0	T2B N0	2a	80	F	PAP
444.0	1b	1b	77	M	SQ
445.0	T1A N2	3a	55	F	AC
446.0	T2A N0	1b	79	M	PAP
447.0	T1A N0	1a	70	F	AC
448.0	T1A N0	1a	67	F	SOL
449.0	T1A N1	2a	73	M	AC
450.0	T2A N0	1b	76	M	ADSQ
451.0	T2A N0	1b	79	F	MUC
452.0	T4 (M) N0	3a	72	F	SOL
453.0	T1A N0	1a	60	M	AC
454.0	T1B N2	3a	79	F	MUC
455.0	T2A N2	3a	69	F	MP
456.0	T1A N0	1a	59	F	AC
457.0	T1B N0	1a	60	F	LPA
458.0	3a	3a	63	M	LCC
459.0	T2A N1	2a	75	F	MP
460.0	T2A N2	3a	77	M	AC
461.0	T1B N0	1a	71	F	ADSQ
462.0	T2A N2	3a	81	M	SIG
463.0	T1A N2	3a	74	M	AC
464.0	2a	2a	64	M	SQ
465.0	T1B N2	3a	82	F	AC
466.0	T2A N0	1b	75	F	LPA
467.0	T1A N0	1a	50	F	MUC
468.0	1b	1b	66	F	SQ
469.0	2a	2a	52	M	SQ
470.0	T1A N0	1a	65	F	MIA
471.0	4	4	83	F	SQ
472.0	T2A N0	1b	62	F	AC
473.0	T1A N0	1a	70	M	LPA
474.0	T1A N0	1a	55	F	MIA
475.0	1A	1a	74	F	SQ
476.0	1B	1b	74	M	SQ
477.0	T2A N0	1b	67	F	SOL
478.0	1A	1a	48	F	LCNEC
479.0	T2A N0	1b	81	M	SOL
480.0	1A	1a	80	F	AC

481.0	T1A N0	1a	72	F	AC
482.0	T2A N0	1b	61	F	AC
483.0	1A	1a	70	F	SQ
484.0	T1BN0	1a	73	F	LPA
485.0	T1AN1	2a	70	F	SOL
486.0	3A	3a	76	M	SQ
487.0	T3N1	3a	62	M	ADSQ
488.0	T1BN1	2a	69	M	AC
489.0	T1A N0	1a	53	M	MUC
490.0	1A	1a	70	M	SQ
491.0	T1BN1	2a	74	F	
493.0	T1A N0	1a	65	M	AC
494.0	1a	1a	70	F	AC
495.0	1a	1a	62	F	AC
496.0	1a	1a	72	F	AC
497.0	T2N1	2a	68	F	SQ
