# Supplementary figures and images for: Early-Stage Lung Adenocarcinoma MDM2 Genomic Amplification Predicts Clinical Outcome and Response to Targeted Therapy
Source: Cancers (Basel). 2022 Jan 29;14(3):708. doi: 10.3390/cancers14030708 (PMC8833784; doi:10.3390/cancers14030708)

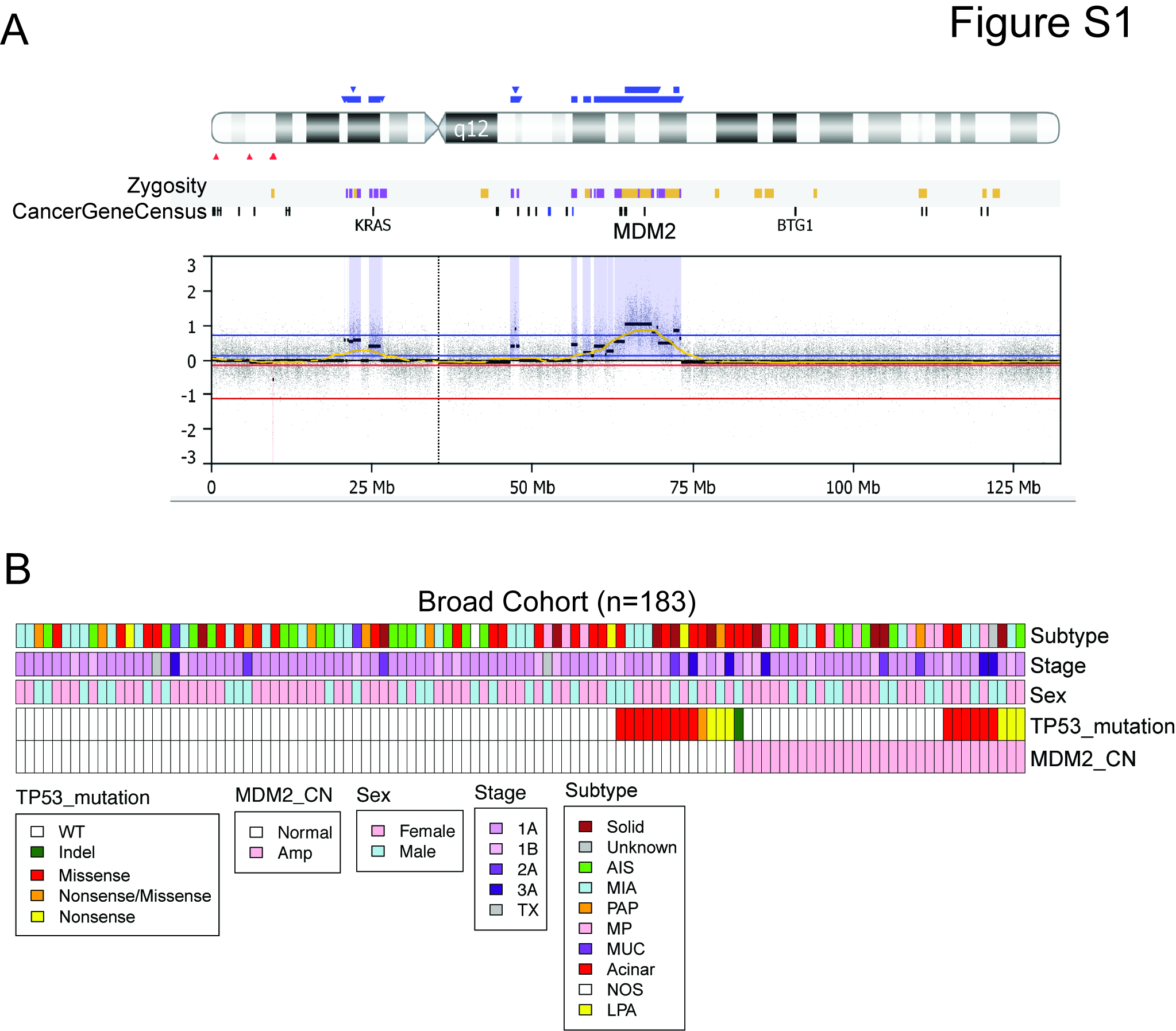

Supplement: Supplementary file 1 [file cancers-14-00708-s001.zip › Fig S1.tif]

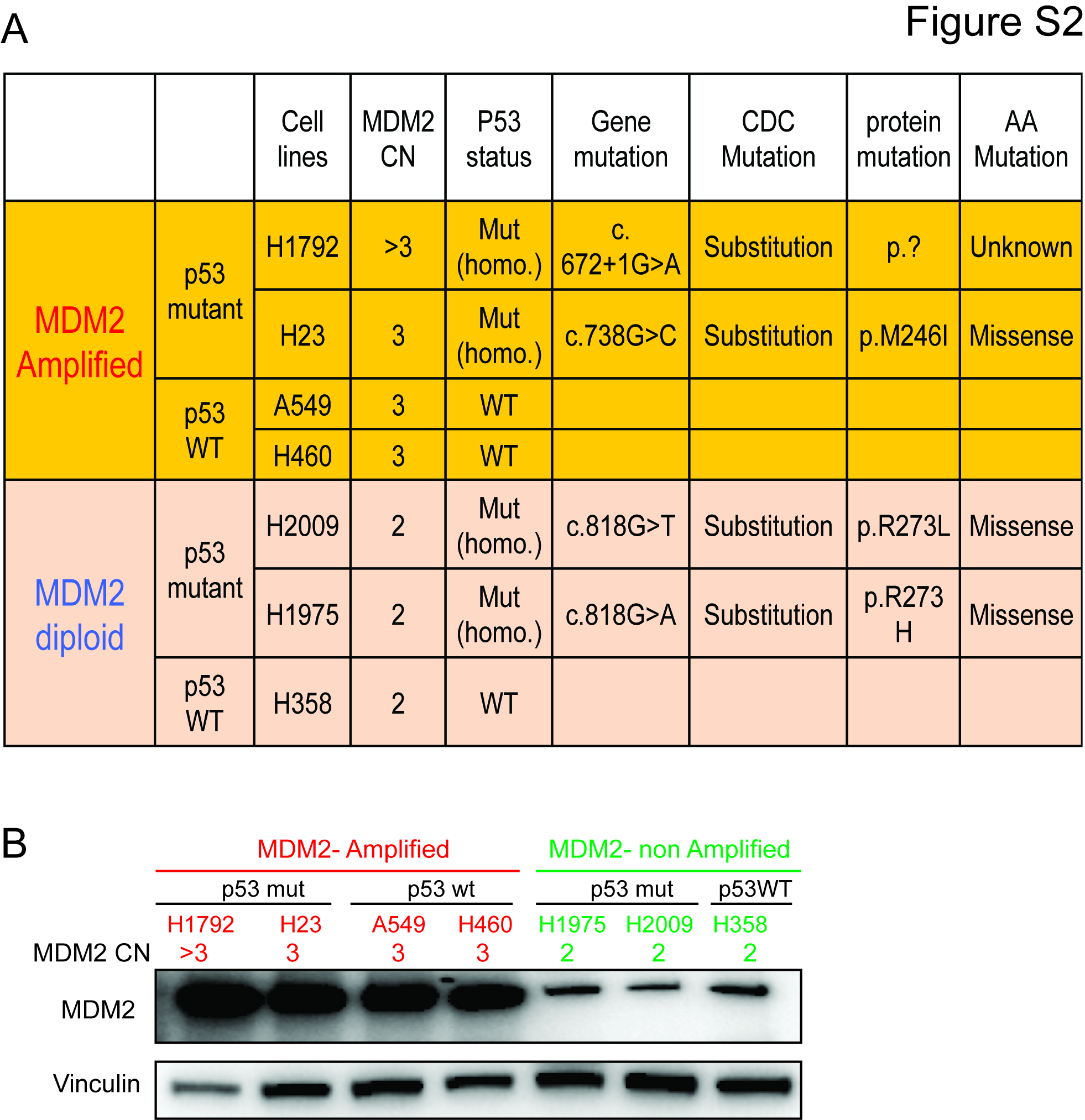

Supplement: Supplementary file 1 [file cancers-14-00708-s001.zip › Fig S2.tif]

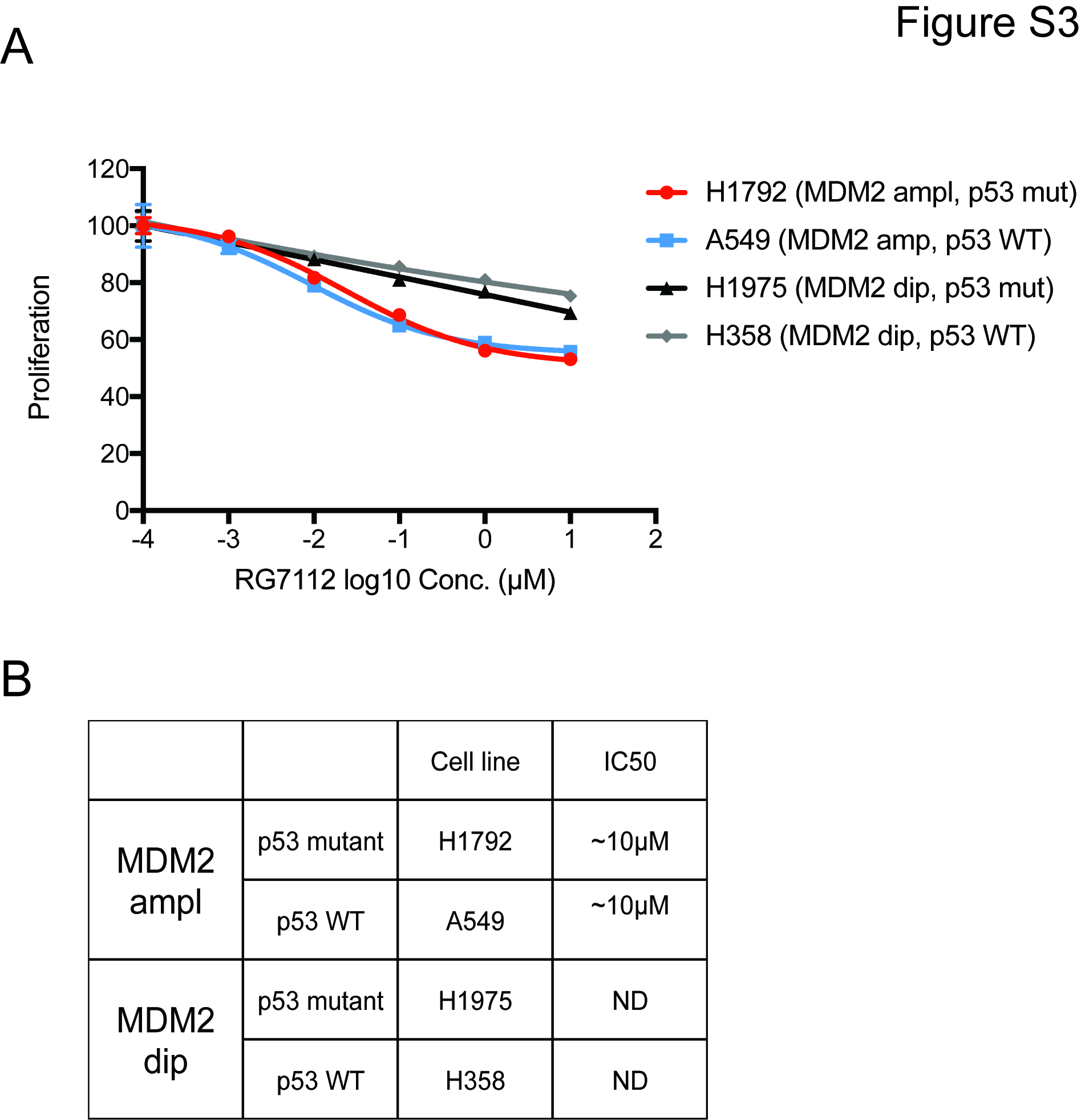

Supplement: Supplementary file 1 [file cancers-14-00708-s001.zip › Fig S3.tif]

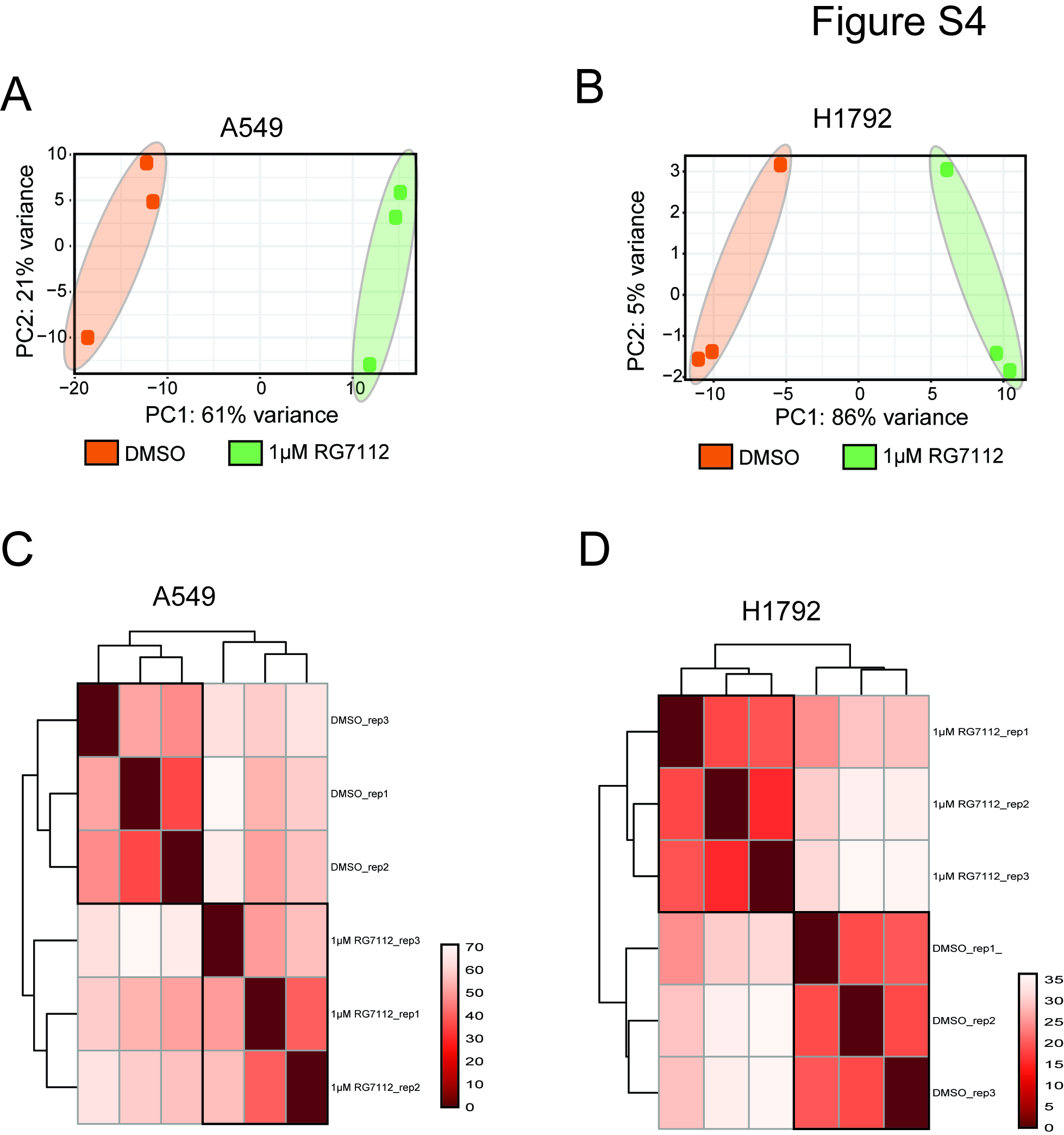

Supplement: Supplementary file 1 [file cancers-14-00708-s001.zip › Fig S4.tif]

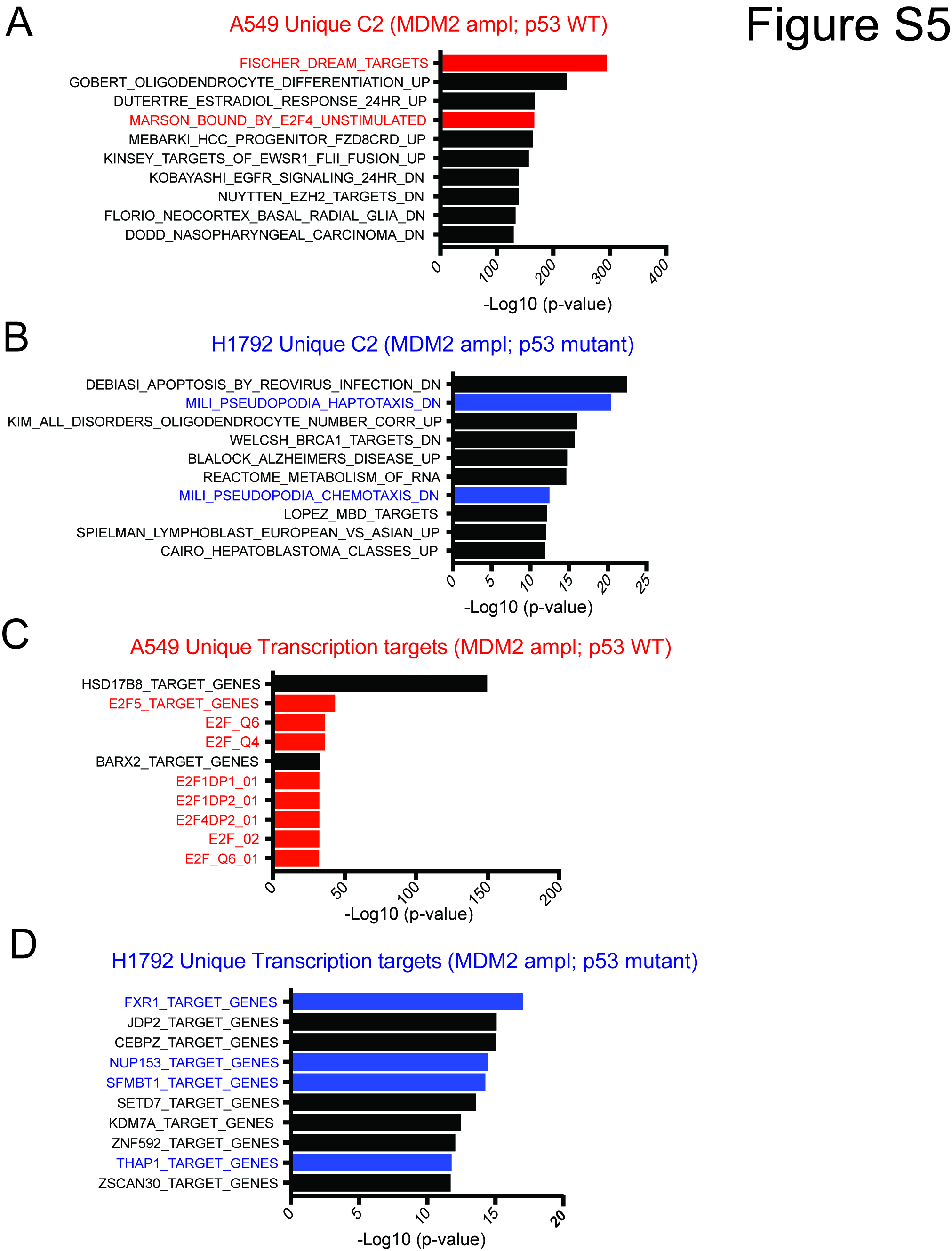

Supplement: Supplementary file 1 [file cancers-14-00708-s001.zip › Fig S5.tif]

A. Whole blot for Fig 5A

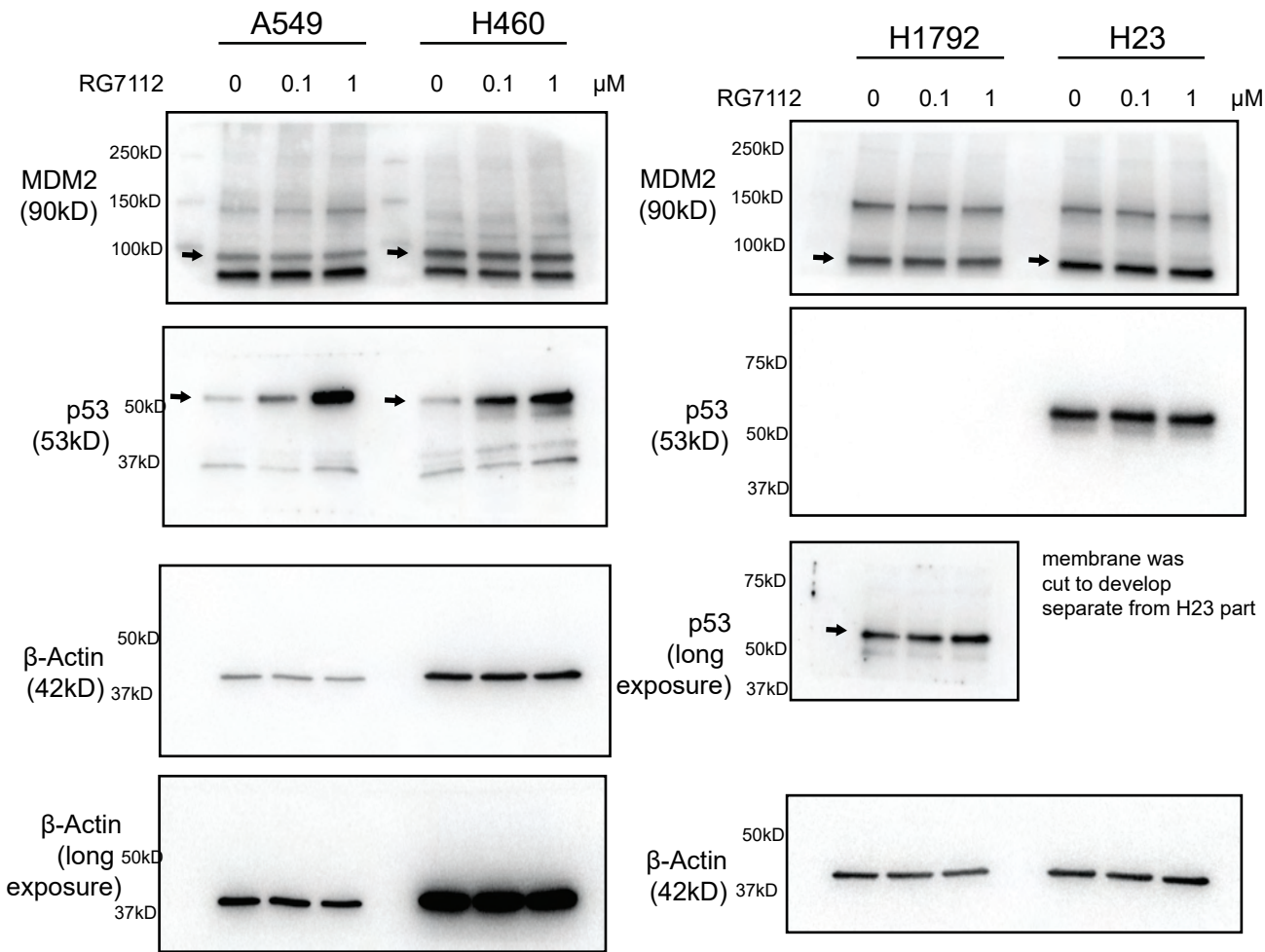

B. Whole blot for Fig 6B

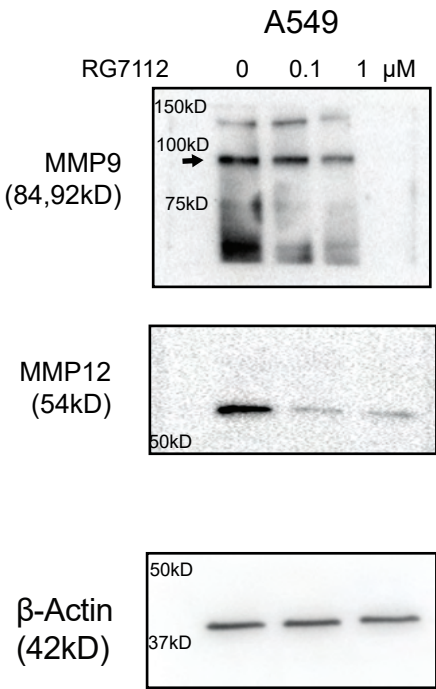

C. Whole blot for Fig 6C

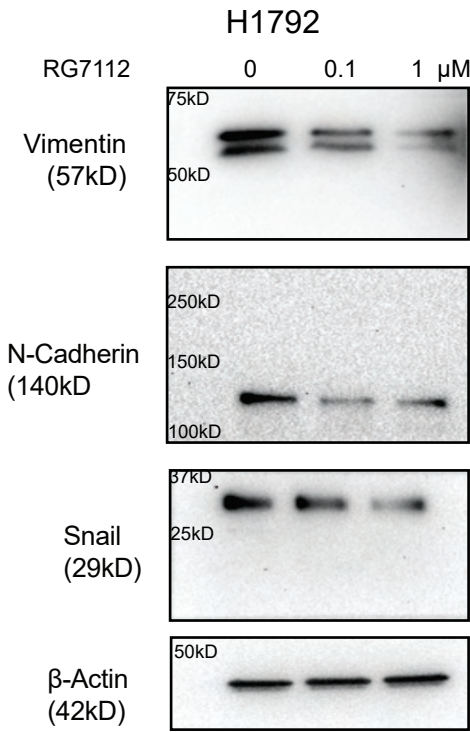

D. Whole blot for Fig S2B

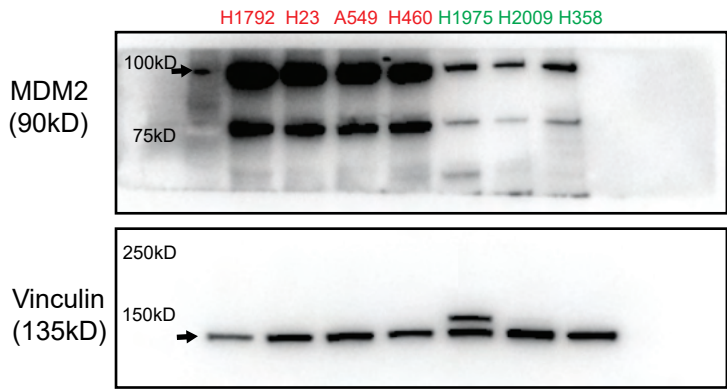

Supplement: Supplementary file 1 [file cancers-14-00708-s001.zip › western.pdf]
